# A sustainable organizational structure to integrate psycho-social stimulation programme into primary health care services in Bangladesh: protocol for a pragmatic cluster randomized controlled trial on scaling up early childhood development activities

**DOI:** 10.1186/s40359-025-02795-w

**Published:** 2025-05-19

**Authors:** S. M. Mulk Uddin Tipu, Sheikh Jamal Hossain, Shams El Arifeen, Syeda Fardina Mehrin, Nur E Salveen, Masuma Kawsir, Mohammad Saiful Alam Bhuiyan, Shamima Shiraji, Mohammed Imrul Hasan, Fahmida Tofail, Jena Derakhshani Hamadani

**Affiliations:** 1 Maternal and Child Health Division, icddrb, Dhaka, Bangladesh; 2 Global Health and Migration Unit, Department of Women’s and Children’s Health, Uppsala University, Uppsala, Sweden; 3 Nutrition Research Division, icddrb, Dhaka, Bangladesh

**Keywords:** Bangladesh, Early Childhood Development, Health system, Malnourished children Psychosocial stimulation

## Abstract

**Background:**

Early childhood development (ECD) in low to middle-income nations has been a pressing concern for the last two to three decades. It is estimated that approximately 250 million children under the age of five are not reaching their full developmental potential due to factors such as poverty, malnutrition, and insufficient home stimulation. It is estimated that in Bangladesh, 44% of the children under 5 years live in poverty and 31% are stunted, and both factors are risks for poor childhood development. We aim to develop a sustainable, scaled ECD programme using an evidence-based curriculum of psychosocial stimulation for disadvantaged Bangladeshi children.

**Methods:**

Approximately 10,000 malnourished children aged 6–24 months will be identified by mid-upper arm circumference (MUAC) < 13.5 cm, assessed by government health staff in over 500 community clinics (CCs) in 21 sub-districts of Hobiganj, Brahmanbaria, Laxmipur and Narsingdi districts. The children and their mothers will participate in the psychosocial stimulation programme that will be delivered fortnightly for one year to small groups at the CCs by health workers. We will train approximately 1,500 health staff in rural CCs to deliver the ECD sessions. We will follow a cascade training model to train personnel at all levels of the primary health care system. A subsample will be evaluated using a cluster randomized controlled trial in 48 CCs (24 intervention and 24 control). From each CC, 10 children aged 6–24 months will be tested before and after the programme. The primary outcome is children’s cognitive development and we will also measure language composite scores on the Bayley Scales of Infant and Toddler Development. Secondary outcomes are children’s growth, maternal knowledge and depressive symptoms, and stimulation in the home. Intention-to-treat analysis will be used to assess intervention effects.

**Discussion:**

There are no existing ECD services for the children under three years delivered by the health care system in Bangladesh. This study will help policy makers to consider incorporating ECD activities into the treatment of malnourished children in rural CCs, under the government’s primary health care system, thereby developing a sustainable structure for improving disadvantaged children’s development. The findings will also provide information to facilitate scaling ECD in the primary health care systems in other low and middle-income countries.

**Trial registration:**

ClinicalTrials.gov Identifier: NCT04093934 (Date: 13 April 2022).

**Supplementary Information:**

The online version contains supplementary material available at 10.1186/s40359-025-02795-w.

## Introduction

Around 250 million children under the age of five in low and middle-income countries (LMICs) fail to realize their complete potential [1] due to poverty, malnutrition and lack of a stimulating environment [2]. In Bangladesh, all the above risks are present and many children are exposed to several risk factors at the same time that can have cumulative effects on their development. Moreover, Bangladesh is one of the most densely populated countries in the world and although the country has had some remarkable improvement in health and education sectors, the nutritional status of the children remains poor. The latest Bangladesh Demographic Health Survey (2017) reported that the estimated prevalence of moderate and severe stunting in children under five were 31% and 9% respectively [3]. A separate study revealed that impoverished, rural Bangladeshi children exhibited a notable cognitive deficit as early as 7 months old, compared to their wealthier counterparts. The deficit continued to widen as the children reached 5 years of age, becoming significant by that point, with factors such as poor growth, maternal education, and lack of home stimulation playing key roles [4].

Undernourished children are thus most at risk of poor development and require additional interventions [8]. In Bangladesh, at least half of the children cannot reach their developmental potential based on proxy indicator of poverty and stunting [6, 7]. Psychosocial stimulation may help mitigate some of the effects on malnutrition on cognitive development (reference), but parenting practices in most LMICs tend to be insufficient and the quality of stimulation in the home is limited with few children having access to toys or books and few parents participating in playing and chatting with them [5]. We have modified the Reach Up and Learn curriculum (http://www.reachupandlearn.com), a comprehensive curriculum integrating psychosocial stimulation and play that was developed and tested among underprivileged children across several studies in Bangladesh [4, 12–15] and other developing countries [5–10, 12]. In all the studies conducted in Bangladesh, we consistently observed significant positive impacts on children’s cognitive and language development [4, 12–15]. We also found that the impact of the intervention on IQ is also sustained at the middle childhood of Bangladeshi children [16].

Our latest research includes two cluster randomized controlled trials, through which we introduced this intervention in 130 rural CCs in Bangladesh [4, 13]. We tailored the curriculum to accommodate to intimate gatherings of mothers and children, comprising either 2 or 4 pairs, who convened biweekly at the clinic over the span of a year. The intervention yielded significant advantages in both the development and behavior of the children involved [5, 7]. To develop a sustainable model, we trained Community Health Care Providers (CHCPs), Health Assistants (HAs) and Family Welfare Assistants (FWAs) who work at CCs. All providers delivered the integrated ECD service from the CCs and were supervised by research staff. In another study, we trained the Health Inspectors (HIs), Assistant Health Inspectors (AHIs), and Family Planning Inspectors (FPIs), who are the supervisors of providers and asked them to train and supervise the CHCP, HA and FWA [17].In the current study, we … Our ultimate plan is to institute a sustainable programme that integrates (ECD) activities into the daily operations of CCs in rural Bangladesh, specifically targeting undernourished children. This will be accomplished through a cascade training model, commencing with district-level staff, extending to sub-district personnel, and ultimately reaching the CCs. The primary outcomes are children’s cognition and language development, and behaviour. Secondary outcomes are children’s growth, maternal knowledge and depressive symptoms and stimulation in the home. The objective of this study is to measure if an integrated psycho-social stimulation programme into primary health care services in Bangladesh will improve children’s development compared to the without intervention when it is designed to scale the study.

We hypothesize that integrating psychosocial stimulation programme into primary health care services in Bangladesh will improve children’s development compared to the control group and can be taken to scale in the country.

## Methods

### Study design

This is a pragmatic cluster randomized control trial with two arms i) intervention and ii) control. We are planning to allocated the intervention randomly to the Unions, a local government unit. We also plan to train front-line health workers (HAs, FWAs and CHCPs) of the control arm when the end-line will be completed.

The cluster randomization was chosen because it would be unethical to assign individual children in each clinic to intervention and control groups. It was also done to avoid contamination and to align with the existing primary health care system of Bangladesh, where everyone can come to the clinic and we can conduct sessions for them accordingly. Logistical feasibility was not an issue.

### Study area

We have selected Hobigonj from the Sylhet Division and Brahmanbaria & Laxmipur from the Chittagong Division based on high prevalence of malnutrition in those areas[18]. There were 31% stunted children in Bangladesh whereas it was 42.4% in Sylhet division in 2018 [19]. In addition, we have selected Narsingdi in the Dhaka Division where we had previously conducted the pilot for this scale-up programme. We aim to target 21 Upazilas in these 4 districts with approximately 500 CCs each enrolling at least 20 children making an approximate total of 10,000 children.

### Inclusion and exclusion criteria

Malnourished children with mid-upper arm circumference (MUAC) < 13.5 cm, aged 6–24 months of both sexes, live within walking distance and children whose parents consent to participate in the programme will be eligible for inclusion, while those with severe malnutrition, disabilities, multiple births, or any congenital abnormalities (although all children with disabilities will attend the intervention session), will not be included in the evaluation sample. Children with severe malnutrition, disability and congenital abnormality will be referred for treatment as required. The health care providers will be trained to identify malnourished children based on their MUAC measurements.

### Procedure

**Fig. 1:**
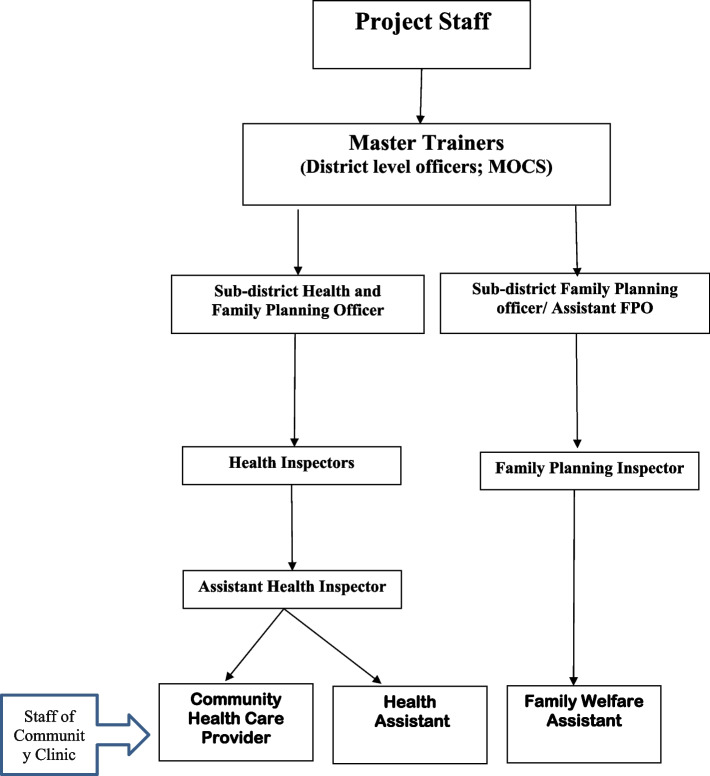
Cascade of Training

We are planning to use a cascade model of ECD training where Medical Officers (MOs) at the Sub-district or District level, trained by the project staff, will in turn, train the supervisors (AHI, HI & FPI). MOs and respective trainer and Upazila Health and Family Planning officer (Upazila Health Manager), together, will select the supervisors, who show the most promise, to train and supervise the providers (CHCP, HA & FWA) (Fig. 1). A trained MO will monitor the cascade model of this training in their respective sub-district. We will also integrate monitoring and supervision mechanisms with the supervisor’s (MO/AHI/HI/FPI) into the regular visits of the CCs. We will deploy only one project staff (recruited for the ECD activity) mainly for coordination. The project staff will also coordinate the activities and conduct coaching during the training sessions if required.

### Intervention

The identified malnourished children and their mothers will be invited by the providers to attend the CCs for fortnightly intervention sessions on psychosocial stimulation for at least one year. The CHCPs mainly work at the CCs for six days a week and they are assisted by HAs and FWAs on two days a week. They will conduct the sessions for groups of up to five mothers and child dyads for 45–60 min. These front-line health workers will plan themselves to conduct sessions. They will identify the malnourished children themselves from the community when they (HAs & FWAs) will work at community or when CHCPs will deliver their regular health care from the CCs. The group curriculum of the Reach-Up and Learn that was already used in Bangladesh [7] will be used for this study with minor modifications. The curriculum incudes songs, age specific toys and picture books, language activities, and nutritional and developmental messages at each session aiming to improve children’s overall development and at the same time raising mothers’ self-esteem and knowledge of child rearing. The Reach Up and Learn is a weekly curriculum for children 6–42 months old [20]. This curriculum can be modified to a fortnightly format. This curriculum introduced an approach delivered by community workers to show parents simple and inexpensive ways of interacting with their young children using homemade toys, books, and conversation. The curriculum was developed based on Bandura’s social-cognitive learning theory [21]. Since this study will be operated by the government providers of the health system, the providers will motivate the caregivers to attend the sessions regularly.

### Evaluation

We will assess the effects of intervention by evaluating a subsample of the children and mothers who participate in the intervention comparing them with children who have not yet participated. The children in the controlled Unions will receive intervention after the end line evaluation has been conducted. Randomization will be at the Union level. A statistician who is not involved with the study will randomize through a computer-generated sequence. The statistician will be provided name of the Unions (local government unit, will be considered as clusters) and then the 48 unions will be randomly allocated to either intervention or control, with 24 unions in each group.

Our goal is to hand over the intervention to government health system and therefore we will plan to develop a system within the government for continuous monitoring and supervision.

### Sample size

Using a cluster randomized controlled trial, with 80% power, 5% level of significance, intra cluster correlation (ICC) of 0.05, 10% attrition rate and 0.33 standard deviation (SD) improvement in cognitive development in the intervention group, a total of 480 children will be required. The improvement of cognitive development was based on the calculation of standardised effect size of 0.33SD and we found this effect in another study in Bangladesh [15].

### Sampling process

The study will be conducted in four districts (21 subdistricts). Primarily, we will randomly select two subdistricts from each district and then six Unions (random selection) from each subdistrict. Randomization will be done at the Union level where 48 Unions will be randomized to intervention and control for the study, and 10 children will be assessed from each CC.

**Fig. 2:**
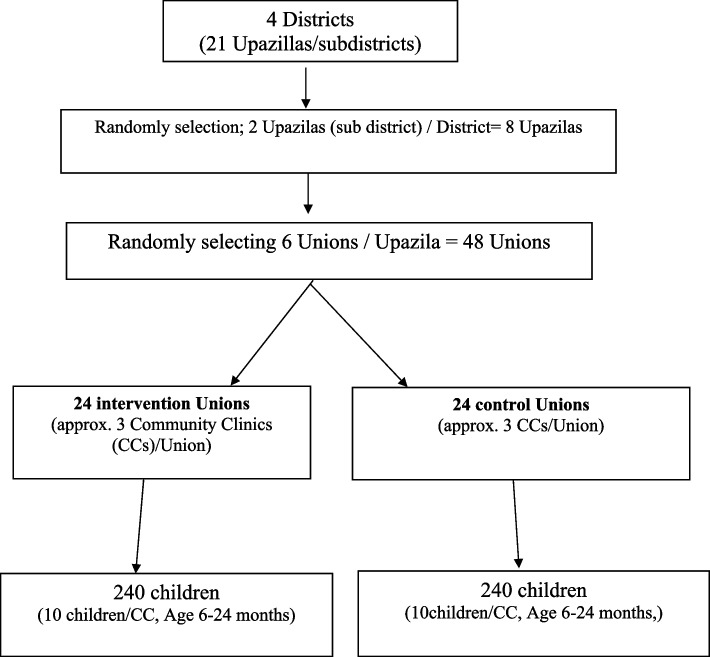
Enrolments of the study participants

Figure 2 shows the selection process of the evaluation from each CC.

### Measures

Bayley Scales of Infant and Toddler Development–third edition (Bayley-III) [22] will be used to assess children’s cognitive and language development, while children’s behaviour will be measured using Wolke’s behaviour ratings[23]. Bayley has been used in several developing countries[14, 24, 25] and the Bangladeshi adapted Bayley scale was used in many settings in Bangladesh [4, 13, 14]. Quality of psychosocial stimulation will be assessed using Family Care Indicators (FCI), which was developed by UNICEF[26] and validated in Bangladesh [27]. This questionnaire was used in several studies in Bangladesh [13, 14, 28]. The mothers/caregivers are asked questions e.g. number of toys and picture books in the house, did the mother/father/any adult had activities such as play with toys with the child or read/sing/tell stories in the last three days etc. Maternal knowledge of child rearing will be measured using a previously developed questionnaire that has been used in several of our studies in Bangladesh [5, 7]. Higher scores of FCI and maternal knowledge indicate better environment for child stimulation and maternal knowledge.

Weight and length/height of the children will be measured using WHO methods and converted to z scores [29]. All the primary and secondary outcomes will be assessed at enrolment and after one year of the intervention. Mothers’ height and weight will be measured at baseline and will be converted to BMI. Families’ socioeconomic status will be assessed using a questionnaire only at baseline. The assessors will be blind to children’s assigned groups (intervention or control), but they may get to know the group during endline assessment while interacting with mothers and children, as the mothers may talk about the intervention they received. We do not expect any adverse effects even due to intervention. Mothers will be provided with the mobile phone number of the Principal Investigator to contact her for any problem or adverse situation during the intervention period.

### Data collection, quality and entry

Data will be collected by trained data collectors having at least a Bachelor’s degree in psychology or social science. The data collectors will be trained on Bayley scales [22].

The interobserver reliability of the Bayley test will be assessed. Only when they reach acceptable standards of reliability will they begin testing. Data will be collected using tablets. All the data will be gathered in a software of icddr,b. The quality of the data will be ensured through observing 10% of the interviews in the field during ongoing data collection. Data quality will also be checked through checking extreme values.

### Data analysis

Data will be analysed using the STATA (version 12.1). The age adjusted composite score of children’s cognitive and language development will be used for analysis as primary outcomes. The children’s cognitive and language development raw score will be calculated through assessment of the children and then these raw scores will be converted into scaled score and finally the scaled score will be converted to composite scores. The guideline of scoring will be followed by Bayley manual. The scores of family care indicators and mothers’ knowledge of child care will be added to calculate a total FCI and a total knowledge score. The higher scores mean higher performance. Families’ socioeconomic status will be assessed using a questionnaire only at baseline that we used previously in several studies in Bangladesh’s context for child development studies only at baseline. We will collect parental age, education, occupation, number of children as background information. We will also collect several assets the families own e.g. mobile phone, television, chairs, tables, motorcycle, domestic animals: goats, cows, hens, etc., to calculate a wealth index. The participants’ background characteristics will be presented in mean (SD) or proportion. Principle component analysis will be followed to present wealth index. To understand if there are any differences at enrolment between participants from the two groups, the background information and outcomes will be presented by groups (intervention vs control) to see if there is any imbalance. Intention-to-treat analysis will be followed to measure the impact of the intervention on primary and secondary outcomes. Primary outcomes are children’s cognitive and language development and the secondary outcomes are child stimulation environment and mothers’ knowledge on child care.

All participants who will be enrolled and will be available in the intervention area after receiving at least one session will be included in the analysis.

Multilevel modelling analysis will be used to assess the impact of the intervention controlling for the confounding factors and clusters. The confounding factors are considered to those variables that show imbalance between intervention and comparison and/or will be correlated with main outcomes. Age, sex of the children and corresponding outcome variable at enrolment and testers’ effect will also be adjusted in the multilevel model. Effect size of the intervention will be reported. Adjustments for multiple testing will also be applied. Subgroup analysis of the data e.g. by mothers’ education, gender of the children etc. will be conducted. A pilot was conducted 5 years prior to this study and the children who participated in the pilot have grown up, so they are not in the age range of the study. Some of the frontline health workers may still be there and we want to know if their prior training brings about any change through the analysis by district. We will also analyse the results by prior and present intervention to assess if there is any difference due to having a prior training.

We will justify the findings through managing missing data analysis by multiple imputation. To address missing data, multiple imputation by chained equations (MICE) will be employed, generating 10 imputed datasets. Rubin’s rules will be used to combine the estimates to obtain unbiased findings.

### Data sharing

Data will be publicly available in an accessible format according to icddr,b data policy. The data set can be made available three years after the end of the study based on request. The findings of the study will be published in peer-reviewed journals. In addition, the preliminary finding will be shared with different stakeholders.

### Consent, harms and confidentiality

We will get written approval from the participants. We will inform the participants that they can withdraw themselves at any time during interview or intervention. We will describe all the details to the participants ensuring that there will not be any harm to them from participating in the study. We will also confirm the participants that the information will be analysed anonymously and the findings will not be presented using participants’ particulars like name and address.

We will refer the disabled children to appropriate place depending on the type of their disability. We will conduct process evaluation and record their experiences and challenges.

### Trial status

Data collection stage.

## Discussion

In Bangladesh, numerous children face the compounded challenges of poverty, malnutrition and limited access to stimulating environments, impeding their development. Extensive evidence underscores that ECD interventions coupled with comprehensive provider training significantly enhance children’s development and behaviour. Consequently, we advocate for nationwide scaling of ECD initiatives to address these pressing issues comprehensively. We documented that the service providers of CCs can provide ECD services when trained and supervised by the project staff [4] and the intervention improves the children’s development at an early age. In this project, we are testing the cascade training model and the service providers will be supervised and monitored by their upper level staff within their regular activities. This study will help develop the sustainable organizational structure needed to integrate an ECD programme for malnourished children into Bangladesh primary health care services to improve children’s development. It will also identify the challenges faced for this scale-up and ways and means to address those challenges.

The strength of this study is to use all health staff MOs, inspectors and service providers in the primary health care system to train, deliver and supervise ECD service from CCs which may be a scalable model. Since we will use a pragmatic cRCT, the findings will be scientifically robust of this scalable mode.

The limitations of the study are that because we are withdrawing the research staff, we will not be able to document the compliance of the intervention especially the use of play materials during intervention, the time taken for each session, the number of children attending the intervention, the regularity of the sessions etc., but we will have to rely on the reporting by the health staff as will happen if the programme goes to scale. In addition, we will be unable to remind the mothers to attend the sessions by phone, as we did previously, because it would be an added expense. Narsingdi in the Dhaka Division, where we previously conducted the pilot for this scale-up programme, may possibly introduce bias into the children’s outcomes in this district. However, we will be cognizant while interpreting the findings of the children of this district. Furthermore, we do not have any social mobilization programme to improve awareness of the communities about ECD programmes, which may weaken the participation in the intervention at CCs of those who require it most.

## Supplementary Information

Supplementary Material 1.

## Data Availability

No datasets were generated or analysed during the current study.

## Abbreviations

CCs: Community Clinics
CHCPs: Community Health Care Providers
FWAs: Family Welfare Assistants
HAs: Health Assistants
HIs: Health Inspectors
AHIs: Assistant Health Inspectors
FPIs: Family Planning Inspectors
ECD: Early Childhood Development
MUAC: Mid-Upper Arm Circumference
BMI: Body Mass Index
FCI: Family Care Indicator
ICC: Intracluster correlation
LMICs: Low- and Middle-Income Countries
WHO: World Health Organization
