# Supplementary material for: A sustainable organizational structure to integrate psycho-social stimulation programme into primary health care services in Bangladesh: protocol for a pragmatic cluster randomized controlled trial on scaling up early childhood development activities
Source: BMC Psychol. 2025 May 19;13:525. doi: 10.1186/s40359-025-02795-w (PMC12090399; doi:10.1186/s40359-025-02795-w)
Supplement: Supplementary file 1 — Supplementary Material 1. [file 40359_2025_2795_MOESM1_ESM.pdf]

icddr,b	**RRC APPLICATION FORM**

RESEARCH PROTOCOL
Number: PR- 19040
Version No. 1.01
Version date: 18-04-2019

FOR OFFICE USE ONLY

RRC Approval:	☐ Yes	☐ No	Date:
ERC Approval:	☐ Yes	☐ No	Date:
AEEC Approval:	☐ Yes	☐ No	Date:
External IRB Approval	☐ Yes	☐ No	Date:
Name of External IRB: ____________________			

Protocol Title:* (maximum 250 characters including space) **To support the government of Bangladesh to integrate an evidence-based programme of psychosocial stimulation into primary health care services and to establish a sustainable organizational structure**

Short Title: (maximum **100** characters including space) Bangladesh Undertaking child Development and Health-system Integration (BUDHI) Project

Key Words:* Psychosocial stimulation, nutrition, primary health care service, ECD, Bayley test, Bangladesh

Name of the Research Division Hosting the Protocol:*

☐ Health Systems and Population Studies Division (HSPSD)
☐ Nutrition and Clinical Services Division (NCSD)
☐ Infectious Diseases Division (IDD)
☒ Maternal and Child Health Division (MCHD)
☐ Laboratory Sciences and Services Division (LSSD)
☐ Other (specify) ____________________

Has the Protocol been Derived from an Activity:* ☒ No ☐ Yes (please provide following information):

Activity No. : Activity Title: PI:
Grant No.: Budget Code: Start Date: End Date:

icddr,b Strategic Priority/ Initiative (SP 2015-8):* (check all that apply)

☐ Reducing maternal and neonatal mortality
☐ Controlling enteric and respiratory infections
☒ Preventing and treating maternal and childhood malnutrition
☐ Detecting and controlling emerging and re-emerging infections
☒ Achieving universal health coverage
☐ Examining the health consequences of climate change
☐ Preventing and treating non-communicable diseases
☐ Others (specify)____________________

Research Phase (4 Ds):* (check all that apply**)**

☐ Discovery
☐ Development
☒ Delivery
☒ Evaluation of Delivery

Anticipated Impact of Research:* (check all that apply and please provide details below**)**

☒ Knowledge Production
☒ Capacity Building
☐ Informing Policy
☒ Health and Health Sector Benefits
☐ Economic Benefits

Please provie details here: The proposal will improve our knowledge about integration of nutrition and ECD activities into the health system. It will develop capacity in the health care professionals at national, district, subdistrict and rural levels as well as in our staff. The policy makers will be informed about the results and can improve the health policy, whereby the health sector will be benefitted.

Which of the Sustainable Development Goal This Protocol Relates to?:* (check all that apply)

☐ 1. End poverty in all its forms everywhere
☐ 2. End hunger, achieve food security and improved nutrition and promote sustainable agriculture
☒ 3. Ensure healthy lives and promote well-being for all at all ages
☒ 4. Ensure inclusive and equitable quality education and promote lifelong learning opportunities for all
☐ 5. Achieve gender equality and empower all women and girls
☐ 6. Ensure availability and sustainable management of water and sanitation for all
☐ 7. Ensure access to affordable, reliable, sustainable and modern energy for all
☐ 8. Promote sustained, inclusive and sustainable economic growth, full and productive employment and decent work for all
☐ 9. Build resilient infrastructure, promote inclusive and sustainable industrialization and foster innovation
☐ 10. Reduce inequality within and among countries
☐ 11. Make cities and human settlements inclusive, safe, resilient and sustainable
☐ 12. Ensure sustainable consumption and production patterns
☐ 13. Take urgent action to combat climate change and its impacts
☐ 14. Conserve and sustainably use the oceans, seas and marine resources for sustainable development
☐ 15. Protect, restore and promote sustainable use of terrestrial ecosystems, sustainably manage forests, combat desertification, and halt and reverse land degradation and halt biodiversity loss
☐ 16. Promote peaceful and inclusive societies for sustainable development, provide access to justice for all and build effective, accountable and inclusive institutions at all levels
☐ 17. Strengthen the means of implementation and revitalize the global partnership for sustainable development

**Does this Protocol Use the Gender Framework:*** (Please visit: http://www.icddrb.net.bd/jahia/Jahia/pid/684 for Gender Alanysis Tool with instructions)	☒ Yes (please complete Gender Analysis Tool)<br>☐ No
If 'no' is the response, its reason(s) in brief:	
**Will this Research Specifically Benefit the Disadvantaged** (economically, socially and/or otherwise**):**	☒ Yes<br>☐ No
**Does this Protocol use Behaviour Change Communication:**	☒ Yes ☐ No
**Principal Investigator (Should be icddr,b staff):*** Sex ☒ Female ☐ Male<br>Dr Jena D Hamadani, Scientist, 01713093849, Ext. 2353, jena@icddrb.org<br>Do you have ethics certification? ☐ No ☒ Yes (please attach in your CV below)<br>Do you have RBM training certification? ☒ No ☐ Yes	Primary Scientific Division of the PI: MCHD
**Co-Principal Investigator(s) Internal**: Sex ☐ Female ☒ Male<br>Dr. Dewan Md. Emdadul Hoque<br>Project Coordinator, Maternal and Neonatal Health<br>Maternal and Child Health Division, icddr,b<br>emdad@icddrb.org, Mob:-01713048530<br>Signature or written consent of Co-PI: ________________<br>Do you have ethics certification? ☐ No ☒ Yes (please attach in your CV below)<br>Do you have RBM training certification? ☐ No ☒ Yes	Primary Scientific Division/ Programme of the Co-PI: MCHD<br>________________<br>Approval of the Respective Senior Director/ Programme Head<br>(Signature)
**Co-Principal Investigator(s) – Internal::** Sex ☒ Female ☐ Male<br>Dr. Fahmida Tofail, Scientist and Consultant Physician, icddr,b, ftofail@icddrb.org, 8860523-32 (2350)<br>Signature or written consent of Co-PI: ________________<br>**Do you have ethics certification? ☐ No ☒ Yes (please attach in your CV below)**<br>**Do you have RBM training certification? ☐ No ☒ Yes**	Primary Scientific Division/ Programme of the Co-PI:: NCSD<br>________________<br>Approval of the Respective Senior Director/ Programme Head<br>(Signature)
**Co-Investigator(s) - Internal:** Sex ☐ Female ☒ Male<br>Dr Shams El Arifeen, Senior Director, 3800, shams@icddrb.org<br>Signature or written consent of Co-I: ________________<br>Do you have ethics certification? ☐ No ☒ Yes<br>Do you have RBM training certification? ☐ No ☒ Yes	Primary Scientific Division/ Programme of the Co-I: MCHD<br>________________<br>Approval of the Respective Senior Director/ Programme Head<br>(Signature)

Co-Investigator(s) - Internal: Sex ☐ Female ☒ Male
Dr Md. Imrul Hasan, Assistant Scientist, 2331, imrul@icddrb.org

Signature or written consent of Co-I: ________________
Do you have ethics certification? ☐ No ☒ Yes

Do you have RBM training certification? ☐ No ☒ Yes

Primary Scientific Division of the Co-I:
MCHD________________
Approval of the Respective Senior Director/ Programme Head

(Signature)

Co-Investigator(s) - Internal: Sex ☒ Female ☐ Male
Ms Fardina Mehrin, Research Investigator, 2331, fardina.mehrin@icddrb.org

Signature or written consent of Co-I: ________________
Do you have ethics certification? ☐ No ☒ Yes

Do you have RBM training certification? ☐ No ☒ Yes

Primary Scientific Division of the Co-I:
MCHD________________
Approval of the Respective Senior Director/ Programme Head
(Signature)

Co-Investigator(s) - Internal: Sex ☒ Female ☐ Male
Ms Shamima Shiraji
Research Investigator, 2331, 01913122399, shamima.shiraj@icddrb.org

Signature or written consent of Co-I: ________________
Do you have ethics certification? ☐ No ☒ Yes
Do you have RBM training certification? ☐ No ☐ Yes

Primary Scientific Division of the Co-I:
MCHD________________
Approval of the Respective Senior Director/ Programme Head

(Signature)

Co-Investigator(s) - Internal: Sex ☐ Female ☒ Male
Mr Sheikh Jamal Hossain:
Assistant Scientist,01712191414,sheikh.jamal@icddrb.org

Signature or written consent of Co-I: ________________
Do you have ethics certification? ☐ No ☒ Yes
Do you have RBM training certification? ☐ No ☒ Yes

Primary Scientific Division of the Co-I:
MCHD________________
Approval of the Respective Senior Director/ Programme Head

(Signature)

Co-Investigator(s) - Internal: Sex ☒ Female ☐ Male
Ms Afroza Hilaly, Research investigator, con:-01711232004, e-mail:-ahilaly@icddrb.org
Signature or written consent of Co-I: ________________
Do you have ethics certification? ☐ No ☒ Yes

Do you have RBM training certification? ☒ No ☐ Yes

Primary Scientific Division of the Co-I:
MCHD________________
Approval of the Respective Senior Director/ Programme Head
(Signature)

Co-Investigator(s) – External: Sex ☒ Female ☐ Male
Prof Sally Grantham McGregor, Institute of Child Health, UCL, sallymcgregor@yahoo.com
Signature or written consent of Co-I: ________________

Co-Investigator(s) – External: Sex ☒ Female ☐ Male
Dr Helen Baker-Henningham, School of Psychology, Bangor University, UK and Senior Lecturer in Child Development, Tropical Medicine Research Institute, University of the West Indies, Mona, Kingston 7, Jamaica, Tel: (1876) 927 2471, helen.henningham@uwimona.edu.jm
Signature or written consent of Co-I: ________________

Collaborating Institute(s): Please provide full official address
Institution # 1

Country	UK
Contact person	Prof Sally Grantham McGregor
Department (including Division, Centre, Unit)	Institute of Child Health
Institution (with official address)	University College London
Directorate (in case of GoB i.e. DGHS)	
Ministry (in case of GoB)	

Institution 2

Country	UK
Contact person	Dr Helen Baker-Henningham
Department (including Division, Centre, Unit)	School of Psychology
Institution (with official address)	Bangor University
Directorate (in case of GoB i.e. DGHS)	
Ministry (in case of GoB)	

Contribution by the Members of the Scientific Team:

Members' Name	Contribution								
	Research idea/ concept	Study design	Protocol writing	Respond to external reviewers' comments	Defending at IRB	Developing data collection Tool(s)	Data Collection	Data analysis/ interpretation of results	Manuscript writing
Dr. Jena Hamadani	☒	☒	☒	☒	☒	☒	☐	☒	☒
Dr. Emdadul Haque	☐	☐	☐	☐	☐	☐	☐	☐	☐
Dr. Fahmida Tofail	☐	☐	☒	☒	☒	☒	☐	☐	☒
Dr. Shams Arifeen	☐	☐	☐	☐	☐	☐	☐	☐	☐
Dr Md Imrul Hasan	☐	☐	☐	☐	☒	☐	☒	☒	☒
Ms Syeda Mehrin	☐	☐	☐	☐	☒	☐	☒	☒	☒
Ms Shamima Shiraji	☐	☐	☐	☐	☒	☐	☒	☒	☒
Mr Sk Hossain	☐	☐	☐	☐	☒	☐	☒	☒	☒
Ms Afroza Hilaly	☐	☐	☐	☐	☒	☐	☒	☒	☒
Prof Sally McGregor	☒	☒	☐	☐	☐	☐	☐	☒	☒
Dr Helen Baker-Henningham	☒	☒	☐	☐	☐	☐	☐	☒	☒

Study Population: Sex, Age, Special Group and Ethnicity

Research Subject:
- ☒ Human
- ☐ Animal
- ☐ Microorganism
- ☐ Other (specify): ______________

Sex:
- ☒ Male
- ☒ Female
- ☐ Transgender

Age:
- ☒ 0 – 4 years
- ☐ 5 – 10 years
- ☐ 11 – 17 years
- ☒ 18 – 64 years
- ☐ 65 +

Special Group:
- ☐ Pregnant Women
- ☐ Fetuses
- ☐ Prisoners
- ☐ Destitutes
- ☐ Service Providers
- ☐ Cognitively Impaired
- ☐ CSW
- ☐ Expatriates
- ☐ Immigrants
- ☐ Refugee
- ☐ Others (specify): _________________

Ethnicity:
- ☒ No ethnic selection (Bangladeshi)
- ☐ Bangalee
- ☐ Tribal group
- ☐ Other (specify): _________________

NOTE: It is icddr.b's policy to include men, women, children and transgender in its research projects involving participation of humans, unless there is strong justification(s) for their exclusion.

Consent Process: (Check all that apply)
- ☒ Written
- ☐ Oral
- ☐ Audio
- ☐ Video
- ☐ None

Language:
- ☒ Bangla
- ☐ English
- ☐ Other (specify: ________________

Project/Study Site: (Check all that apply)
- ☐ Chakaria
- ☐ Bandarban
- ☐ Dhaka Hospital
- ☐ Kamalapur Field Site/HDSS
- ☐ Mirpur (Dhaka)
- ☐ Matlab DSS Area
- ☐ Matlab non-DSS Area
- ☐ Matlab Hospital
- ☐ Mirzapur
- ☐ Bianibazar (Sylhet)
- ☐ Kanaighat (Sylhet)
- ☐ Jakigonj (Sylhet)
- ☐ Other community in Dhaka
  Name: ___________________________________
- ☒ Other sites in Bangladesh
  Name: Sylhet & Rangpur Divisions
- ☐ Multi-national Study
  Name of the country_______________________

Project/Study Type: (Check all that apply)

- ☐ Case Control Study
- ☐ Clinical Trial (Hospital/Clinic/Field)*
- ☒ Community-based Trial/Intervention
- ☐ Cross Sectional Survey
- ☐ Family Follow-up Study
- ☐ Longitudinal Study (cohort or follow-up)
- ☐ Meta-analysis
- ☐ Programme Evaluation
- ☐ Programme (Umbrella Project)
- ☐ Prophylactic Trial
- ☐ Record Review
- ☐ Secondary Data Analysis
  Protocol No. of Data Source: _____________
- ☐ Surveillance/Monitoring
- ☐ Systematic Review
- ☐ Other (specify): _________________________

***Note**: International Committee of Medical Journal Editors (ICMJE) defines Clinical Trial as "*Any research project that prospectively assigns human participants to intervention and comparison groups to study the cause-and-effect relationship between a medical intervention and a health outcome*".

PI of the RRC- and ERC-approved Clinical Trials should provide necessary information to IRB Secretariat (Research Administration) for registration and uploading into relevant websites (usually at the https://register.clinicaltrials.gov/). They should also provide relevant information to the IRB Secretariat in the event of amendment/modification after their approval by RRC and ERC.

Biological Specimen:

a) Will the biological specimen be stored for future use?	☐ Yes ☐ No ☒ Not applicable
b) If the response is 'yes', how long the specimens will be preserved?	__________ years
c) What types of tests will be carried out with the preserved specimens?	NA
d) Will the consent be obtained from the study participants for use of the preserved specimen for other initiative(s) unrelated to this study, without their re-consent?	☐ Yes ☐ No ☒ Not applicable
e) Will the specimens be shipped to other country/ countries? If yes, name of institution(s) and country/countries.	☐ Yes ☐ No ☒ Not applicable<br>Name ________________________________
f) If shipped to another country, will the surplus/unused specimen be returned to icddr,b? If the response is 'no', then the surplus/unused specimen must be destroyed.	☐ Yes ☐ No ☒ Not applicable
g) Who will be the custodian of the specimen at icddr,b?	NA
h) Who will be the custodian of the specimen when shipped outside Bangladesh?	NA
i) Who will be the owner(s) of the specimens?	
j) Has a MoU been signed with regards to collection, storage, use and ownership of specimen? If the response is 'yes', please attach a copy of the MoU. If the response is 'no', appropriate justification should be provided for not signing a MoU.	☐ Yes ☐ No ☒ Not applicable

Proposed Sample Size for evaluation:

Sub-group (Name of subgroup e.g. Men, Women) and Number

Name	Number	Name	Number
(1) Undernourished intervention	388	(3)	
(2) Undernourished control	388	(4)	
		**Total sample size**	776

Determination of Risk: Does the Research Involve (Check all that apply**)**

- ☐ Human exposure to radioactive agents?
- ☐ Foetal tissue or abortus?
- ☐ Investigational new device?
  Specify: ___________________
- ☐ Existing data available from Co-investigator?
- ☐ Human exposure to infectious agents?
- ☐ Investigational new drug?
- ☐ Existing data available via public archives/sources?
- ☐ Pathological or diagnostic clinical specimen only?
- ☒ Observation of public behaviour?
- ☐ New treatment regime?

Will the information be recorded in such a manner that study participants can be identified from the information directly or through identifiers linked to the study participants?	Yes ☒ No ☐
Does the research deal with sensitive aspects of the study participants' sexual behaviour, alcohol use or illegal conduct such as drug use?	Yes ☐ No ☒
**Could information on study participants, if available to people outside of the research team:**	
a) Place them at risk of criminal or civil liability?	Yes ☐ No ☒
b) Damage their financial standing, reputation or employability, or social rejection, or lead to stigma, divorce etc.?	Yes ☐ No ☒

Do you consider this research: (check one)

☐ Greater than minimal risk	☒ No more than minimal risk	☐ Only part of the diagnostic test

Note: Minimal Risk: The probability and the magnitude of the anticipated harm or discomfort to participants is not greater than those ordinarily encountered in daily life or during the performance of routine physical, psychological examinations or tests, e.g. the risk of drawing a small amount of blood from a healthy individual for research purposes is no greater than when the same is performed for routine management of patients.

**Risk Group of Infectious Agent and Use of Recombinant DNA**	
a) Will specimens containing infectious agent be collected?	☐ Yes ☐ No ☒ Not applicable
b) Will the study involve amplification by culture of infectious agents?	☐ Yes ☐ No ☒ Not applicable
c) If response to questions (a) and/or (b) is 'yes', to which Risk Group (RG) does the agent(s) belong? (Please visit http://www.icddrb.net.bd/jahia/Jahia/pid/684 to review list of microorganism by Risk Group)	☐ RG1 ☐ RG2 ☐ RG3 ☐ RG4
d) Does the study involve experiments with recombinant DNA?	☐ Yes ☐ No ☒ Not applicable

Does the study involve any biohazards materials/agents or microorganisms of risk group 2, 3, or 4 (GR2, GR-3 or GR4)?

☐ Yes ☒ No

[If the response is 'yes'] I, (print name of the PI) affirm that we will use the standard icddr,b laboratory procedures for biosafety of the hazardous materials/agents or microorganisms in the conduction of the study.

______________________________ ______________

Signature of the Principal Investigator **Date**

Dissemination Plan: [please explicitly describe the plans for dissemination, including how the research findings would be shared with stakeholders, identifying them if known, and the mechanism to be used; anticipated type of publication (working papers, internal (institutional) publication, international publications, international conferences/seminars/workshops/ agencies. [Check all that are applicable]

**Dissemination type**	**Response**		**Description (if the response is a yes)**
Seminar for icddr,b scientists/ staff	☐ No	☒ Yes	
Internal publication	☒ No	☐ Yes	
Working paper	☒ No	☐ Yes	
Sharing with GoB (e.g. DGHS/ Ministry, others)	☐ No	☒ Yes	
Sharing with national NGOs	☐ No	☒ Yes	
Presentation at national workshop/ seminar	☐ No	☒ Yes	
Presentation at international workshop/ conference	☐ No	☒ Yes	
Peer-reviewed publication	☐ No	☒ Yes	
Sharing with international agencies	☒ No	☐ Yes	
Sharing with donors	☐ No	☒ Yes	
Policy brief	☒ No	☐ Yes	

`**Funding:**

Is the protocol fully funded?	☒ Yes	☐ No
If the answer is yes, please provide sponsor(s)'s name	1. MoWCA, GoB	
	2. Grand Challenges Canada	
Is the protocol partially funded?	☐ Yes ☐ No	☒ NA
If the answer is yes, please provide sponsor(s)'s name	1.	
	2.	

If fund has not been identified:

Is the proposal being submitted for funding?	☐ Yes ☐ No	☒ NA
If yes, name of the funding agency	1.	
	2.	

Conflict of interest:

Do any of the participating investigators and/or member(s) of their immediate families have an equity relationship (e.g. stockholder) with the sponsor of the project or manufacturer and/or owner of the test product or device to be studied or serve as a consultant to any of the above?

☒ No ☐ Yes (please submit a written statement of disclosure to the Executive Director, icddr,b)

Proposed Budget:

Dates of Proposed Period of Support **Cost Required for the Budget Period ($)**

(Day, Month, Year - DD/MM/YY)

Beginning Date : 01/05/2019

End Date : 30/04/2021

Years	Direct Cost	Indirect Cost	Total Cost
**Year-1 (GCC)**	383004	0	383004
**Year-1 (GoB)**	624501	87430	711931
**Year-2 (GCC)**	380640	0	380640
**Year-2 (GoB)**	223970	31356	255326
**Total**	1612115	118786	1730901

Indirect cost of 14% is in GoB budget, while in-built overhead is @11% for GoB and 25% for GCC.

Certification by the Principal Investigator:

I certify that the statements herein are true, complete and accurate to the best of my knowledge. I am aware that any false, fictitious, or fraudulent statements or claims may subject me to criminal, civil, or administrative penalties. I agree to accept the responsibility for the scientific conduct of the project and to provide the required progress reports including updating protocol information in the NAVISION if a grant is awarded as a result of this application.

I also certify that I have read icddr,b Data Policies and understand the PIs' responsibilities related to archival and sharing of research data, and will remain fully compliant to the Policies. (Note: The Data Policies can be found here: http://www.icddrb.org/who-we-are/data-policies)

______________ ______________

Signature of PI **Date**

Approval of the Project by the Division Director of the Applicant:

The above-mentioned project has been discussed and reviewed at the Division level.

Dr Shams El Arifeen ______________

Name of the Division Director Signature Date of Approval

Table of Contents

RRC APPLICATION FORM 1
Project Summary 9
Hypothesis to be tested: 9
Specific Objectives: 9
Background of the Project including Preliminary Observations: 10
Research Design and Methods 11
Sample Size Calculation and Outcome (Primary and Secondary) Variable(s) 13
Data Analysis 13
Data Safety Monitoring Plan (DSMP) 13
Ethical Assurance for Protection of Human rights 13
Use of Animals 13
Collaborative Arrangements 14
Facilities Available 14
Literature Cited 14
Budget 144
Other Support 19
Biography of the Investigators 20
Format for Consent Form 33
Check-List 388

☒ Check here if appendix is included

Project Summary

[The summary, within a word limit of 300, should be stand alone and be fully understandable.]

Principal Investigator: Dr. Jena Derakhshani Hamadani

Research Protocol Title: To support the government of Bangladesh to integrate an evidence-based programme of psychosocial stimulation and nutritional counselling into primary health care services and to establish a sustainable organizational structure

Proposed start date: 1st May 2019 Estimated end date: 30th April 2021

Background (brief):

a. Burden: Global child development: Approximately 250 million children under 5 years of age in developing countries do not reach their full potential due to poverty, malnutrition and lack of a stimulating environment. It is estimated that in Bangladesh 44% of the population live below the international poverty line, and 36% of children under 5 years are stunted. Bangladeshi children showed a significant cognitive deficit as early as 7 months of age compared to more affluent children and the deficit grew bigger as children reached 5 years of age.
b. Knowledge gap: Several studies in developing countries have shown benefits of early childhood interventions to development of under-5 children. The curriculum of early childhood intervention 'Reach up' has been adapted for Bangladesh and used in 6 trials in Bangladesh. All the projects found consistent significant benefits on the developmental outcomes of children. Two recent trials were conducted in community clinics (CCs), integrating with Govt primary health service and there is a need to determine if the intervention can be taken to scale.

c. Relevance:

Bangladesh Govt acknowledges the need for improving children's development and has agreed to collaborate and implement early childhood development (ECD) activities at large scale in addition to funding it.

Hypothesis (if any): We hypothesize that it is feasible to train GoB staff at District, Sub-district, Union and CC levels and integrate ECD activities in CCs in Bangladesh and thereby improve malnourished children's development after a year of intervention.

Objectives:

1. To establish an organizational structure for the programme to be sustainable
2. To establish a mechanism for sustainability at central, district and sub-district levels: including training , supervision, monitoring and reporting
3. To assess the impact on maternal knowledge and depressive symptoms, stimulation in the home and child growth, cognition and language in a subsample

Methods: Malnourished children aged 6-24 months will be identified using mid-arm upper circumference (MUAC) by Govt. Health staff in 13 sub-districts of Sylhet and 8 sub-districts of Rangpur division. We target to include 554 Community clinics in the programme and train approx. 1600 CC staff to deliver the parenting session. We will conduct a small case-control study with a subsample of the children.

Outcome measures/variables: The main outcomes are coverage, compliance and fidelity of the programme. In addition, children's cognitive and language development and behaviour will be assessed in a sub-sample.

Description of the Research Project

Hypothesis to be tested:

In a hypothesis testing research proposal, briefly mention the hypothesis to be tested and provide the scientific basis of the hypothesis, critically examining the observations leading to the formulation of the hypothesis.

Does this research proposal involve testing of hypothesis: ☐ No ☒ Yes (describe below)

We hypothesize that it is feasible to train GoB staff at District, Sub-district, Union and CC levels and integrate ECD activities in CCs in Bangladesh and thereby improve malnourished children's development after a year of intervention.

Specific Objectives:

Describe the specific objectives of the proposed study. State the specific parameters, gender aspects, biological functions, rates, and processes that will be assessed by specific methods.

General objective is to establish a sustainable programme whereby ECD activities are integrated into the routine activities of CCs in rural Bangladesh for undernourished children and national, district, sub-district, and union level trainers are developed to train clinic staff.

The specific objectives are:

1. To establish a mechanism for sustainability at central, district and sub-district levels: including training , supervision, monitoring and reporting
2. To assess the feasibility of the sustainable model including:
   a) *Coverage*: Assess number of children participating in the programme

b) *Compliance* of inspectors and clinic staff

i. Attendance at training,

ii. % of sessions run,

iii. % of sessions observed by inspectors

c) *Fidelity*:

i. % of inspector reports correctly filled in

ii. % of sessions meeting acceptable standards

3. To assess the impact on maternal knowledge and depressive symptoms, stimulation in the home and child growth, cognition and language in a subsample

Background of the Project including Preliminary Observations:

Provide scientific validity of the hypothesis based on background information of the proposed study and discuss previous works on the research topic, including information on sex, gender and diversity (ethnicity, SES) by citing specific references. Critically analyze available knowledge and discuss the questions and gaps in the knowledge that need to be filled to achieve the proposed aims. If there is no sufficient information on the subject, indicate the need to develop new knowledge.

Global child development: Approximately 250 million children under 5 years of age in developing countries do not reach their full potential due to poverty, malnutrition and lack of a stimulating environment (Black et al. 2016).
In Bangladesh, all the above risks are prevalent and many children are exposed to several risk factors at a time that have cumulative effects on their development. Almost half of the children under 5 years of age suffer from some form of malnutrition. It is also a low middle income country and one of the most densely populated in the world. A recent study showed that poor Bangladeshi children showed a significant cognitive deficit as early as 7 months of age compared to more affluent children and the deficit grew bigger as children reached 5 years of age (Figure 1).

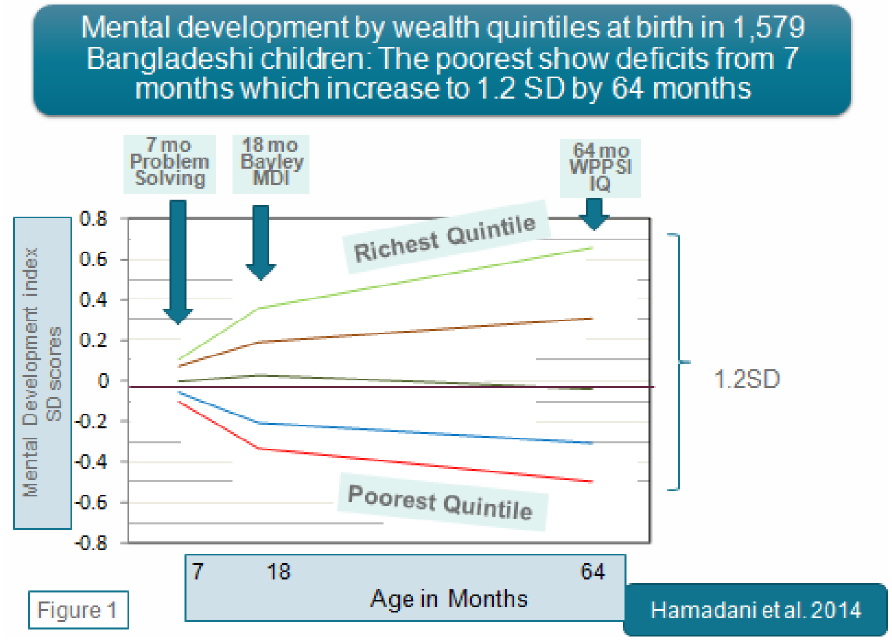

Figure 1

In early childhood, parenting practices tend to be poor and the quality of stimulation in the home is very limited with few children having access to toys or books and parents playing and chatting with them. At least half the children in Bangladesh are therefore disadvantaged and among them, undernourished children are most at risk of poor development and require additional interventions. The Child development unit (CDU) at icddr,b in Bangladesh has modified the Reach-Up curriculum, a comprehensive curriculum of psychosocial stimulation and play and tested it for underprivileged children in six studies (Hamadani et al. 2006;Nahar et al. 2009;2012;Tofail et al. 2013; Tofail et al. (2)- in preparation). In all the studies we consistently found significant benefits to children's development and behaviour.
More recently we conducted two large cluster randomized controlled trials in 130 community clinics (CCs) where we adapted the same curriculum for small groups of mothers and children to be run at 65 CCs (Hamadani et al. 2019; Mehrin et al. in preparation). In these studies, we introduced the early childhood development (ECD) and nutritional counselling programme for malnourished children aged 6-24 months and compared them to children in 65 control CCs. Mothers with their children attended sessions at the clinic every two weeks for one year. In the first study mothers attended in pairs (Hamadani et al. 2019) and in the second one they attended in small groups (Mehrin et al. in preparation). To develop a sustainable model, we trained the Government's staff working in CCs to provide ECD intervention for undernourished children as part of their routine work. The studies showed substantial and comprehensive benefits to children's development and behaviour.

Qualitative research found most mothers and providers were satisfied with the programme and were willing to continue if the programme continued further.

Research Design and Methods

Describe the research design and methods and procedures to be used in achieving the specific aims of the research project. If applicable, mention the type of personal protective equipment (PPE), use of aerosol confinement, and the need for the use BSL2 or BSL3 laboratory for different part of the intended research in the methods.. Define the study population with inclusion and exclusion criteria, the sampling design, list the important outcome and exposure variables, describe the data collection methods/tools, and include any follow-up plans if applicable. Justify the scientific validity of the methodological approach (biomedical, social, gender, or environmental).Also, discuss the limitations and difficulties of the proposed procedures and sufficiently justify the use of them.

This proposal will cover the first phase of the intervention implementation and we will set up a work plan with all necessary supports for the second phase (e.g. manualised curriculum, instruments for observing visits, training manual, and system for producing play materials as well as trained master trainers), incorporating any lessons learnt from the 1st stage.

1st stage: Implementation trial

Inclusion criteria:

- Moderately and severely malnourished children aged 6-24 mo of both sexes and whose parents consent to participate in the programme.
- Mid-upper arm circumference (MUAC)<12.5cm will be used to define malnutrition
- Routes of identification of eligible children:
  A. At sub-district level
     a. Children attending integrated management of childhood illnesses (IMCI) corners
     b. Children attending treatment centres for severe acute malnutrition
  B. At *Union* level
     a. Children attending family welfare centres (FWCs)
  C. At village level
     a. Children attending immunization centres
     b. Children attending community clinics (CCs) for illness or immunization

Exclusion criteria:

- Children with MUAC>12.5 cm
- Those whose parents do not consent to participate
- Children with disability, multiple births or any congenital abnormality will be included in the intervention but excluded from the evaluation sample.

Simultaneous with the training, we will identify the eligible children.

Selection of area:

We have selected Sylhet and Rangpur Divisions based on high prevalence of malnutrition in those areas.
From each Division, we have selected 2 districts; hence we will be working in 4 districts. We have dropped the City corporation sub-districts and will have 13 sub-districts in Sylhet (Hobigonj & Moulvi Bazar) and 8 sub-districts in Rangpur (Rangpur & Thakurgaon). We therefore aim to target 21 sub-districts with a total number of 554 CCs. These CCs are supervised by approximately 418 supervisors (Family Planning Inspectors, Health Inspectors and Assistant Health Inspectors) and are run by approximately 1600 health staff in rural areas, all of whom will receive training. We expect to enrol approximately 40 children per clinic.

Procedure

We will work with the Ministry of Health and Family Welfare (MOHFW) and the Ministry of Women and Children Affairs (MOWCA); the two leading ministries for ECD in Bangladesh. We will recruit and train 3 Master Trainers at national level. Through a cascade of training they will train and develop a pool of trainers of trainers (TOT) in each of the 4 districts in consultation with the 'Civil Surgeon' (CS) who is the highest health administrative personnel in a district. We have identified possible personnel at district level, based on their time availability, skills, remaining length of service and educational level, but there may be different personnel at each district, therefore we will consult with each CS before selecting persons for training. The possible candidates at this level are:

1. District health education Officers (DHEO) (1 senior and 1 junior)
2. Public Health Nurse
3. Assistant Director, Clinical Contraceptive (ADCC)
4. Medical officer under CS (MOCS)
5. Medical Officer, Disease Control (MODC)
6. District Shishu (child) Academy Officer

In each district we will have a pool of 5-6 TOTs from the above, based on the recommendation of the CS, however, all of them including the CS will be given a one-day orientation on the programme.

Training:
In previous studies we have conducted several training programmes for Government and NGOs and have expertise in training trainers. Training for the present study will follow a cascade model from Master Trainers (MT) down to clinic staff (Figure 2).

Training of trainers:
Training will be most extensive at the district level for the TOTs and will include the following:

- Curriculum of ECD activities and nutrition education (5 days)
- Training and mentoring procedures including observing play sessions, managerial and facilitation capacity (1-2 days) for monitoring of training by TOTs and program implementation by health staff in the CCs.

The MTs will each go to one of the districts and train 5 or 6 district TOTs. The district TOTs will be trained in a single group, but there will be hands-on training or individual coaching in order for everyone to practice what is required for such training.

Figure 2. Cascade of training

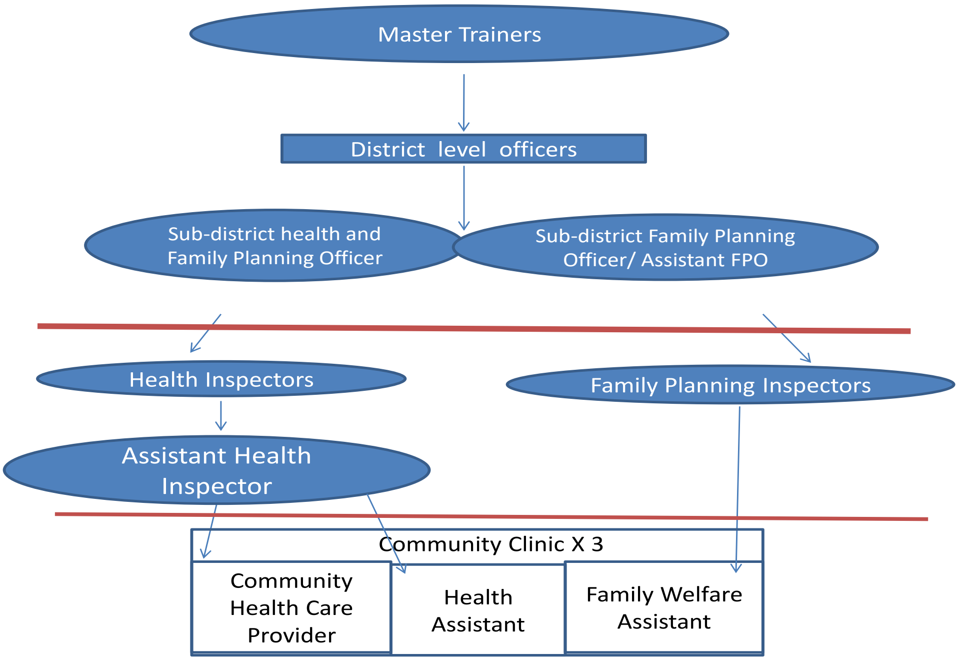

The district TOTs will then be given a schedule to train the next level of TOT at the sub-district level and will be coached by the MTs until they reach acceptable standards.
For the sub-district level of TOT we will consult with the sub-district health and family planning officer and sub-district family planning officer and identify 8 TOTs among the health officers and inspectors in the sub-district. We will have varying number of TOTs in each district, based on the number of sub-districts. The district TOTs will start training at sub-district level in 2-3 phases based on the number of trainees. Our MTs will accompany and observe the training to ensure quality and coach as necessary as the sub-district trainers are trained, they will plan a schedule for the training of the CHCPs, HAs and FWAs in each sub-district. We will request the district TOTs to be present at their training sessions to again ensure the standard of the training. This will result in a high level of training provided to field staff and reduce dilution of the programme.
The research team will pilot the use of training materials before the actual training to ensure its suitability and user-friendliness and to identify any challenges that require handling to ensure that we can hand over the training to the govt. trainers. All these challenges will be recorded and considered for the future training programmes. Each training batch will consist of 15-20 trainees according to the number of trainers in each sub-district.
Training of the clinic workers: The final level of training will be training of the CHCPs, HAs and FWAs and will comprise of:

1. The curriculum of psychosocial stimulation and nutrition

2. Relating to mothers and children and empowering mothers
3. Procedures in conducting group sessions
4. Reporting procedures
5. Placing requisitions, storing and maintaining toys and books at the CC.

Sample Size Calculation and Outcome (Primary and Secondary) Variable(s)

Clearly mention your assumptions. List the power and precision desired. Describe the optimal conditions to attain the sample size. Justify the sample size that is deemed sufficient to achieve the specific aims.

Sample size for the evaluation sample: is calculated using a matched case-control design and effect size of 0.3 SD. We will select 10 children from 40 intervention and 40 control CCs. The control CCs will be the waitlisted CCs in the neighbouring districts of the same Divisions, where intervention has not started yet. The undernourished children will be identified by the research staff and asked to participate in the study. There will be 640 children required for the evaluation. We will add 25% to the sample size to account for losses during the study. Therefore, a sample of 800, 400 in each group will be sufficient to evaluate the study.

Outcome measures

1. Stimulation in the home measured by the Family care indicators (FCI)
2. Maternal knowledge of child rearing
3. Bayley Scales of Infant and Toddler Development-III: language and cognitive scales
4. Wolke's behaviour ratings of children during the Bayley test
5. Anthropometry (weight, length/height, head circumference)

Data Analysis

Describe plans for data analysis, including stratification by sex, gender and diversity. Indicate whether data will be analysed by the investigators themselves or by other professionals. Specify what statistical software packages will be used and if the study is blinded, when the code will be opened. For clinical trials, indicate if interim data analysis will be required to determine further course of the study.

Data will be checked for normality. The monitoring sample will be analysed using frequency distribution of coverage and attendance. The quality of visits will be quantified using a questionnaire and analyzed for fidelity and association with other factors.
Multilevel modelling analysis will be used for intention to treat analysis of the evaluation sample. As the groups are not randomised they may differ on important characteristics we will therefore use propensity score matching using baseline data on the children, families and communities.

Data Safety Monitoring Plan (DSMP)

All clinical investigations (research protocols testing biomedical and/or behavioural intervention(s)) should include the Data and Safety Monitoring Plan (DSMP). The purpose of DSMP is to provide a framework for appropriate oversight and monitoring of the conduct of clinical trials to ensure the safety of participants and the validity and integrity of the data. It involves involvement of all investigators in periodic assessments of data quality and timeliness, participant recruitment, accrual and retention, participant risk versus benefit, performance of trial sites, and other factors that can affect study outcome.

N/A

Ethical Assurance for Protection of Human rights

Describe the justifications for conducting this research in human participants. If the study needs observations on sick individuals, provide sufficient reasons for using them. Indicate how participants' rights will be protected, and if there would be benefit or risk to each participants of the study. Discuss the ethical issues related to biomedical and social research for employing special procedures, such as invasive procedures in sick children, use of isotopes or any other hazardous materials, or social questionnaires relating to individual privacy. Discuss procedures safeguarding participants from injuries resulting from study procedures and/or interventions, whether physical, financial or social in nature. [Please see Guidelines]

Approval of the Research and Ethical Review Committees of icddr,b will be sought. This study will be associated with minimal risks. There are no known risks associated with play stimulation, cognitive and language testing or behavioural observations. Informed written consent will be obtained from the mothers of the participating children for stimulation and psychological examination. Purpose of the study, study precedures, requirements of participation, risks and benefits involved in participation and rights not to participate or wthdraw from the study at anytime will be clearly explained. Mother will be invited to pre-set centres with the child for Bayley test and small snacks and travel cost will be provided. All information collected will be stored under lock and key and kept in a secure place under the close supervision of the Principle Investigator of this study. Data will be kept confidential. Consent papers will be signed in presence of a third person as a witness and one copy of the signed consent will be given to participant mother. The severely malnourished children and those who may be ill will be referred to nearby hospital or health centre through community clinics for further management.

Use of Animals

Describe if and the type and species of animals to be used in the study. Justify with reasons the use of particular animal species in the research and the compliance of the animal ethical guidelines for conducting the proposed procedures.

NA

Collaborative Arrangements

Describe if this study involves any scientific, administrative, fiscal, or programmatic arrangements with other national or international organizations or individuals. Indicate the nature and extent of collaboration and include a letter of agreement between the applicant or his/her organization and the collaborating organization.

This study will be a collaboration of Child Development and Community Mental Health, MCHD at icddr,b, Ministry of Health and Family Welfare, Ministry of Women and Children Affairs, Institute of Child Health, UCL and School of Psychology, Bangor University, UK.

Facilities Available

Describe the availability of physical facilities at site of conduction of the study. If applicable, describe the use of Biosafety Level 2 and/or 3 laboratory facilities. For clinical and laboratory-based studies, indicate the provision of hospital and other types of adequate patient care and laboratory support services. Identify the laboratory facilities and major equipment that will be required for the study. For field studies, describe the field area including its size, population, and means of communications plus field management plans specifying gender considerations for community and for research team members.

The members of the team have developed training manuals and worked extensively on implementation of psychosocial stimulation in different conditions in Bangladesh, India, Jamaica, Brazil, Columbia and other places. FCI is already validated by the Child Development Unit (Hamadani et al. 2010). If any participating child gets sick due to any reason he/she will be referred to local health centres.

A memorandum of understanding will be signed with the Ministry of Health and the CC Trust in Bangladesh to allow using their facilities and involving their staffs.

Literature Cited

Identify all cited references to published literature in the text by number in parentheses. List all cited references sequentially as they appear in the text. For unpublished references, provide complete information in the text and do not include them in the list of Literature Cited. There is no page limit for this section, however, exercise judgment in assessing the "standard" length.

Black, M. M., Walker, S. P., Fernald, L. C. H., Andersen, C. T., DiGirolamo, A. M., Lu, C., et al. Lancet Early Childhood Development Series Steering, C. (2017). Early childhood development coming of age: science through the life course. *Lancet*, *389*(10064), 77-90. doi: 10.1016/S0140-6736(16)31389-7

Hamadani, J. D., Tofail, F., Huda, S. N., Alam, D. S., Ridout, D. A., Attanasio, O., & Grantham-McGregor, S. M. (2014). Cognitive deficit and poverty in the first 5 years of childhood in Bangladesh. *Pediatrics*, *134*(4), e1001-1008. doi: 10.1542/peds.2014-0694

Hamadani JD, Mehrin SF, Tofail F, Hasan MI, Huda SN, Baker-Henningham H, Ridout DA, Grantham-McGregor SM. Integrating an early childhood development programme into the Bangladeshi Primary Health Care Services: A cluster randomised trial. February 2019. Lancet Glob Health 2019; 7: e366–75.
DOI: 10.1016/S2214-109X(18)30535-7.

Hamadani, J. D., Huda, S. N., Khatun, F., & Grantham-McGregor, S. M. (2006). Psychosocial stimulation improves the development of undernourished children in rural Bangladesh. *J Nutr*, *136*(10), 2645-2652.

Nahar, B., Hossain, M. I., Hamadani, J. D., Ahmed, T., Huda, S. N., Grantham-McGregor, S. M., & Persson, L. A. (2012). Effects of a community-based approach of food and psychosocial stimulation on growth and development of severely malnourished children in Bangladesh: a randomised trial. *Eur J Clin Nutr*, *66*(6), 701-709. doi: 10.1038/ejcn.2012.13

Nahar, B., Hamadani, J. D., Ahmed, T., Tofail, F., Rahman, A., Huda, S. N., & Grantham-McGregor, S. M. (2009). Effects of psychosocial stimulation on growth and development of severely malnourished children in a nutrition unit in Bangladesh. *Eur J Clin Nutr*, *63*(6), 725-731. doi: 10.1038/ejcn.2008.44

Tofail, F., Hamadani, J. D., Mehrin, F., Ridout, D. A., Huda, S. N., & Grantham-McGregor, S. M. (2013). Psychosocial stimulation benefits development in nonanemic children but not in anemic, iron-deficient children. *J Nutr*, *143*(6), 885-893. doi: 10.3945/jn.112.160473

Budget [Please add]

Budget Justification Form

As per Finance SLA, please ensure the budget approval process don't take more than 3 days BGT- 133/2019 (R)

Project Title : Strengthening Bangladesh Babies Brains (SBBB)

Grant : New — [x] New — [ ] Existing :: GR# — New Project:: Yes / No — BC#

Donor Name: : Grand Challenges Canada (GCC), Toronto, Ontario, Canada

Main Donor Name : Grand Challenges Canada (GCC), Toronto, Ontario, Canada

Budget Period : 21 Months

PI Name: : Dr. Jena Derakhshani Hamadani

PI's Email & Extn No : Jena@icddrb.org; Ext-2353 — Division Short Name: MCHD

Focal person cell & Extn No: : Ms. Afroza Hilaly — *Study Site Matlab: Yes | No

Budgeted Amount : **CAD 1,000,000 / USD 763,644**

Approving Officer	Justification/Comments	Status		Signature
Principal Investigator/ (PI/FPI)	Donor does not allow indirect cost, therefore the 25% indirect cost has been included in the budget as in-built cost. Grnad Chanllenges Canada dose not allow HVAC and rent either.			[signature]
Head, MHRC (For Matlab based study only)				
Finance Manager/ Assigned Officer	Project period	Yes ●	No ○	[signature] 19.03.19 Md. Rezaur Rahman Manager, Finance MCHD, icddr,b
	Employee CTC	Yes ●	No ○	
	Employee FTE/Time Allocation	Yes ●	No ○	
	Previous Actual Expenditure	Yes ○	No ●	
	Prior approved budget	Yes ○	No ●	
	Additional Amounts	Yes ○	No ●	
	Inflation Adjustment 40%	Yes ●	No ○	
	Activity/Protocol	Yes ○	No ●	
	Budget Soft copy	Yes ●	No ○	
	Other Operating Expenses	Yes ●	No ○	
	Capex	Yes ●	No ○	
	FX rate	Yes ○	No ●	
	Donor communication (email / draft agreement)	Yes ●	No ○	
	Formula and Arithmetic	Yes ●	No ○	
	Audit fees	Yes ○	No ●	
	Overhead Rate	Yes ○	No ●	
	Built in OH 25%	Yes ●	No ○	
	Budget Narrative in line with Budget	Yes ○	No ●	
	Other Comments:			
Senior Budget Coordinator	**Comments:** Direct GH 2019. NonThematic cost $152299F Salary support of DF 24953F, FM $34251, Head RSLD $17570, HR Mng $32147F Head RD $43378F. May be approved. [signature] 19 MAR 19			
Senior Manager, B&P	**Comments:**			[signature] 19.3.19
Finance Analyst	OH/Indirect cost	Yes ○	No ✓	[signature] 20/03/19
	Built in Overhead cost 25%	Yes ✓	No ○	
	Rent and HVAC cost	Yes ○	No ✓	
	IRB cost	Yes ✓	No ○	
	Other support service (ITD) cost	Yes ✓	No ○	
	**Comments:** May be approved.			
Controller, Finance				[signature] 20.3.19
Director, Finance				[signature] 20 MAR 2019

* Please open a separate Budget Code for Built in OH and FPI will be the Director, Finance of this Budget Code.

** All Matlab based upcoming projects (i.e. projects that use any Matlab staff and facilities) budget (new & revision) will have to be reviewed and signed by Head, MHRC before sending to Finance department to make sure that enough funds are budgeted for Matlab core facilities.

RECEIVED 19 MAR 2019 icddr,b Finance

External use restricted

Project Title: Strengthening Bangladesh Babies Brains (SBBB)

PI: Dr Jena Hamadani

Donor: Grand Challenges Canada (GCC)

Duration: 21 months

Year: 2019-2020

Items name	Level	Rate	Number	Effort Y-1	PM Y-1	Yr-1(USD)	Yr-1 (CAD)	Rate	Effort Y-2	PM-2	Yr-2(USD)	Yr-2 (CAD)	Total (USD)	Total (CAD)
		2019 - a	b	c	d	e=(a*b*c*d)	f	2020 - g	h	i	j=(b*g*h*i)	k	l=e+j	
**Personnel**														
PI-Dr Jena Jamadani	P4/5	16,071	1	22%	8	28,285	37,039	16,875	21%	12	42,929	56,216	71,214	93,255
Co-PI-Dr Fahmida Tofail	NOD/7	5,463	1	5%	8	2,063	2,701	6,009	5%	12	3,606	4,722	5,668	7,423
Co-PI-Dr Dewan Md Emdadul Hoque	NOD/7	5,463	1	29%	8	12,805	16,769	6,009	30%	12	21,273	27,857	34,078	44,626
Finance Director	P5/12	24,226	1	5%	8	9,690	12,690	25,437	5%	12	15,262	19,986	24,953	32,676
Finance Manager	NOA/3	2,308	1	70%	8	12,925	16,925	2,539	70%	12	21,326	27,927	34,251	44,852
HR Manager	NOA/5	2,308	1	66%	8	12,131	15,885	2,539	66%	12	20,016	26,211	32,147	42,096
Head, Regulatory and Legal Affairs (N10285)	NOE-2	8,232	1	10%	8	6,704	8,779	9,055	10%	12	10,866	14,229	17,570	23,009
Head, Research Administration	NOE-11	8,072	1	25%	8	16,367	21,432	8,879	25%	12	27,012	35,372	43,378	56,804
Sr. Admn Officer (MCHD)	GS6	1,306	1	82%	8	8,556	11,204	1,437	82%	12	14,102	18,466	22,658	29,670
Office Manager (MCHD)	NOA/8	2,560	1	28%	8	5,866	7,681	2,816	28%	12	9,293	12,169	15,159	19,850
Finance Assistant (Ferdush)	GS4 - 3	816	1	30%	6	1,469	1,923	898	30%	12	3,231	4,231	4,700	6,155
Co-I Assistant Scientist (imrul)	NOB/8	3,188	1	5%	8	1,285	1,683	3,507	5%	12	2,033	2,662	3,318	4,345
Co-I Assistant Scientist (jamal)	NOB/3	2,655	1	5%	8	1,041	1,363	2,921	5%	12	1,643	2,151	2,683	3,514
Liaison member	NOB/3	2,447	1	100%	8	19,576	25,635	2,692	100%	12	32,300	42,298	51,876	67,933
Master Trainers (RI)	NOA	1,972	1	100%	8	15,776	20,659	2,169	100%	9	19,523	25,565	35,299	46,224
Co-I Research Investigator (Training and Quality): Dina	NOA/6	2,392	1	50%	8	9,568	12,529	2,631	50%	10	13,156	17,228	22,724	29,757
Research Investigator (field management) Keya	NOA/4	2,224	1	50%	8	8,896	11,649	2,446	50%	11	13,455	17,620	22,351	29,269
Research Investigator (Sumi)	NOA/4	2,224	1	50%	8	8,896	11,649	2,446	50%	11	13,455	17,620	22,351	29,269
Senior Research Officers (training & quality)	GS6 - CSA	801	1	50%	8	3,204	4,196	881	50%	10	4,406	5,769	7,610	9,965
Field Research Officers (training & quality)	GS5- CSA	641	2	100%	6	7,692	10,073	705	100%	10	14,102	18,467	21,794	28,539
Field Research Officer	GS5-CSA	641	1	100%	6	3,846	5,036	705	100%	2	1,410	1,847	5,256	6,883
Field Research Assistant	GS/3-(CSA)	419	6	100%	6	15,066	19,729	460	100%	2	5,524	7,234	20,590	26,963
Bayley Tester (FRA)	GS/3-(CSA)	419	2	100%	6	5,022	6,576	460	100%	5	4,604	6,028	9,626	12,605
Senior Field Assistants	GS2-CSA	348	2	100%	8	5,566	7,289	383	100%	12	9,185	12,027	14,751	19,317
Data management Supervisor	GS4/11	1,038	1	100%	8	8,304	10,874	1,142	100%	12	13,702	17,942	22,006	28,817
Office/Field attendent	GS2/2-FT	535	1	100%	8	4,280	5,605	589	100%	12	7,062	9,248	11,342	14,852
Field Organizer (Office)	SP level	140	3	100%	8	3,348	4,384	153	100%	12	5,524	7,234	8,872	11,618
**Subtotal**						**238,227**	**311,961**	**106,783**			**349,998**	**458,326**	**588,225**	**770,287**
**Consultants**														
Consutant_Community Clinic	Consolidated	300	1	100%	6	1,800	2,357	330	100%	6	1,980	2,593	3,780	4,950
Consultant_Sally		700	1	100%	2	1,400	1,833	770	100%	2	1,540	2,017	2,940	3,850
Consultant_Helen		7,856	1	8%	6	3,535	4,629	8,642	8%	6	3,889	5,092	7,424	9,722
Subtotal						**6,735**	**8,820**	**9,742**			**7,409**	**9,702**	**14,144**	**18,522**
Training	**Participants & trainers**	**days**	**perdium,**	**batches**	**Miscellenious(secretariat, venue**									
International meeting/conference	4	5	350	0%		-	-		100%		7,000	9,167	7,000	9,167
Joint supervisory and monitorin visit	5	3	200	100%		3,000	3,929				3,000	3,929	6,000	7,857
District level training (1 district in 1 batch)	10	5	30.5	2		3,050	3,994					-	3,050	3,994
Upazilla level training (3upazilla in 1 training)	10	5	15	3		2,250	2,946					-	2,250	2,946
Provider level training (Basic training,	10	6	13	39		30,420	39,835					-	30,420	39,835
Training for recruitment and enrollment	20	1	12	16		3,840	5,029					-	3,840	5,029
Refresher training (upazilla level-6 months after)	10	1	14	9		1,260	1,650					-	1,260	1,650
Refresher training (Provider level)	20	1	13	21		5,460	7,150					-	5,460	7,150
Orientation meeting with national level officer	20	1	20	1		400	524					-	400	524
Orientation meeting with logistic personnel at upzilla	20	1	12	2		480	629					-	480	629
**Sub Total**						**50,160**	**65,685**				**10,000**	**13,095**	**60,160**	**78,780**
**Travel Cost**														
Ineternational travel	4	2,000	4		1	8,000	10,476				8,000	10,476	16,000	20,952
Travel (Central level)		1,450	1		11	15,950	20,887				15,950	20,887	31,900	41,773
Travel local level		1,433	1		11	15,763	20,642				15,763	20,642	31,526	41,284
**Subtotal (travel)**						**39,713**	**52,005**				**39,713**	**52,005**	**79,426**	104,009
**Direct Supplies and services**														
Printing of forms (manuals, reporting forms & others)						5,000	6,548					-	5,000	6,548
Office supplies						1,781	2,332				1,500	1,964	3,281	4,297
Stimulation material						5,000	6,548				-	-	5,000	6,548
**Subtotal (Direct supply & service)**						**11,781**	**15,427**				**1,500**	**1,964**	**13,281**	**17,392**
**Equipments**														
Computer and laptop		1,100	3			3,300	4,321				-	-	3,300	4,321
Printer 1		598	1			598	783					-	598	783
**Subtotal (Equipment)**						**3,898**	**5,104**				-	-	**3,898**	**5,104**
**Other Research costs**														
IRB Clearence						1,510	1,977					-	1,510	1,977
Launching of the project and Dissemination of study findings, seminer, conference						3,000	3,929				-	-	**3,000**	**3,929**
**Subtotal (Other research)**						**4,510**	**5,906**				-	-	**4,510**	5,906
**Total Budget**						**355,024**	**464,908**				**408,620**	**535,092**	**763,644**	**1,000,000**

19.03.19

Md. Rezaur Rahman
Manager, Finance
MCHD, icddr,b

19 MAR 19

19.3.19

Project Title: Strengthening Bangladesh Babies Brains (SBBB)

PI: Dr Jena Hamadani

Donor: Grand Challenges Canada (GCC)

Duration: 21 months

Year: 2019-2020

Items name	Level	Rate	Number	Effort Y-1	PM Y-1	Yr-1(USD)	Yr-1 (CAD)	Rate	Effort Y-2	PM-2	Yr-2(USD)	Yr-2 (CAD)	Total (USD)	Total (CAD)
		2019 - a	b	c	d	e=(a*b*c*d)	f	2020 - g	h	i	j=(b*g*h*i)	k	l=e+j	
**Personnel**														
PI-Dr Jena Jamadani	P4/5	16,071	1	22%	8	28,285	37,039	16,875	21%	12	42,929	56,216	71,214	93,255
Co-PI-Dr Fahmida Tofail	NOD/7	5,463	1	5%	8	2,063	2,701	6,009	5%	12	3,606	4,722	5,668	7,423
Co-PI-Dr Dewan Md Emdadul Hoque	NOD/7	5,463	1	29%	8	12,805	16,769	6,009	30%	12	21,273	27,857	34,078	44,626
Finance Director	P5/12	24,226	1	5%	8	9,690	12,690	25,437	5%	12	15,262	19,986	24,953	32,676
Finance Manager	NOA/3	2,308	1	70%	8	12,925	16,925	2,539	70%	12	21,326	27,927	34,251	44,852
HR Manager	NOA/5	2,308	1	66%	8	12,131	15,885	2,539	66%	12	20,016	26,211	32,147	42,096
Head, Regulatory and Legal Affairs (N10285)	NOE-2	8,232	1	10%	8	6,704	8,779	9,055	10%	12	10,866	14,229	17,570	23,009
Head, Research Administration	NOE-11	8,072	1	25%	8	16,367	21,432	8,879	25%	12	27,012	35,372	43,378	56,804
Sr. Admn Officer (MCHD)	GS6	1,306	1	82%	8	8,556	11,204	1,437	82%	12	14,102	18,466	22,658	29,670
Office Manager (MCHD)	NOA/8	2,560	1	28%	8	5,866	7,681	2,816	28%	12	9,293	12,169	15,159	19,850
Finance Assistant (Ferdush)	GS4 - 3	816	1	30%	6	1,469	1,923	898	30%	12	3,231	4,231	4,700	6,155
Co-I Assistant Scientist (imrul)	NOB/8	3,188	1	5%	8	1,285	1,683	3,507	5%	12	2,033	2,662	3,318	4,345
Co-I Assistant Scientist (jamal)	NOB/3	2,655	1	5%	8	1,041	1,363	2,921	5%	12	1,643	2,151	2,683	3,514
Liaison member	NOB/3	2,447	1	100%	8	19,576	25,635	2,692	100%	12	32,300	42,298	51,876	67,933
Master Trainers (RI)	NOA	1,972	1	100%	8	15,776	20,659	2,169	100%	9	19,523	25,565	35,299	46,224
Co-I Research Investigator (Training and Quality): Dina	NOA/6	2,392	1	50%	8	9,568	12,529	2,631	50%	10	13,156	17,228	22,724	29,757
Research Investigator (field management) Keya	NOA/4	2,224	1	50%	8	8,896	11,649	2,446	50%	11	13,455	17,620	22,351	29,269
Research Investigator (Sumi)	NOA/4	2,224	1	50%	8	8,896	11,649	2,446	50%	11	13,455	17,620	22,351	29,269
Senior Research Officers (training & quality)	GS6 - CSA	801	1	50%	8	3,204	4,196	881	50%	10	4,406	5,769	7,610	9,965
Field Research Officers (training & quality)	GS5- CSA	641	2	100%	6	7,692	10,073	705	100%	10	14,102	18,467	21,794	28,539
Field Research Officer	GS5-CSA	641	1	100%	6	3,846	5,036	705	100%	2	1,410	1,847	5,256	6,883
Field Research Assistant	GS/3-(CSA)	419	6	100%	6	15,066	19,729	460	100%	2	5,524	7,234	20,590	26,963
Bayley Tester (FRA)	GS/3-(CSA)	419	2	100%	6	5,022	6,576	460	100%	5	4,604	6,028	9,626	12,605
Senior Field Assistants	GS2-CSA	348	2	100%	8	5,566	7,289	383	100%	12	9,185	12,027	14,751	19,317
Data management Supervisor	GS4/11	1,038	1	100%	8	8,304	10,874	1,142	100%	12	13,702	17,942	22,006	28,817
Office/Field attendent	GS2/2-FT	535	1	100%	8	4,280	5,605	589	100%	12	7,062	9,248	11,342	14,852
Field Organizer (Office)	SP level	140	3	100%	8	3,348	4,384	153	100%	12	5,524	7,234	8,872	11,618
**Subtotal**						**238,227**	**311,961**	**106,783**			**349,998**	**458,326**	**588,225**	**770,287**
**Consultants**														
Consutant_Community Clinic	Consolidated	300	1	100%	6	1,800	2,357	330	100%	6	1,980	2,593	3,780	4,950
Consultant_Sally		700	1	100%	2	1,400	1,833	770	100%	2	1,540	2,017	2,940	3,850
Consultant_Helen		7,856	1	8%	6	3,535	4,629	8,642	8%	6	3,889	5,092	7,424	9,722
Subtotal						**6,735**	**8,820**	**9,742**			**7,409**	**9,702**	**14,144**	**18,522**
Training	**Participants & trainers**	**days**	**perdium,**	**batches**	**Miscellenious(secretariat, venue**									
International meeting/conference	4	5	350	0%		-	-		100%		7,000	9,167	7,000	9,167
Joint supervisory and monitorin visit	5	3	200	100%		3,000	3,929				3,000	3,929	6,000	7,857
District level training (1 district in 1 batch)	10	5	30.5	2		3,050	3,994					-	3,050	3,994
Upazilla level training (3upazilla in 1 training)	10	5	15	3		2,250	2,946					-	2,250	2,946
Provider level training (Basic training,	10	6	13	39		30,420	39,835					-	30,420	39,835
Training for recruitment and enrollment	20	1	12	16		3,840	5,029					-	3,840	5,029
Refresher training (upazilla level-6 months after)	10	1	14	9		1,260	1,650					-	1,260	1,650
Refresher training (Provider level)	20	1	13	21		5,460	7,150					-	5,460	7,150
Orientation meeting with national level officer	20	1	20	1		400	524					-	400	524
Orientation meeting with logistic personnel at upzilla	20	1	12	2		480	629					-	480	629
**Sub Total**						**50,160**	**65,685**				**10,000**	**13,095**	**60,160**	**78,780**
**Travel Cost**														
Ineternational travel	4	2,000	4		1	8,000	10,476				8,000	10,476	16,000	20,952
Travel (Central level)		1,450	1		11	15,950	20,887				15,950	20,887	31,900	41,773
Travel local level		1,433	1		11	15,763	20,642				15,763	20,642	31,526	41,284
**Subtotal (travel)**						**39,713**	**52,005**				**39,713**	**52,005**	**79,426**	104,009
**Direct Supplies and services**														
Printing of forms (manuals, reporting forms & others)						5,000	6,548					-	5,000	6,548
Office supplies						1,781	2,332				1,500	1,964	3,281	4,297
Stimulation material						5,000	6,548				-	-	5,000	6,548
**Subtotal (Direct supply & service)**						**11,781**	**15,427**				**1,500**	**1,964**	**13,281**	**17,392**
**Equipments**														
Computer and laptop		1,100	3			3,300	4,321				-	-	3,300	4,321
Printer 1		598	1			598	783					-	598	783
**Subtotal (Equipment)**						**3,898**	**5,104**				-	-	**3,898**	**5,104**
**Other Research costs**														
IRB Clearence						1,510	1,977					-	1,510	1,977
Launching of the project and Dissemination of study findings, seminer, conference						3,000	3,929				-	-	**3,000**	**3,929**
**Subtotal (Other research)**						**4,510**	**5,906**				-	-	**4,510**	5,906
**Total Budget**						**355,024**	**464,908**				**408,620**	**535,092**	**763,644**	**1,000,000**

OANDA

Date	Rate
4/2/2019	1.30951
USD	CAD
763,644	1,000,000

Built in OH	152,299
DC	611,345
OH%	25%

Project budget currency:	Click to Select Currency
Exchange rate (project budget currency to CAD):	1.00000

https://www.oanda.com/currency/converter/

1.1 Remuneration - funding recipient's employees

Click here to go to the Budget Guidance

2019-2020

Item of expenditure (name & project role)<br><br>Personnel	Year 1		Year 2		Total Local Currency			Total CAD		
	GCC	Match Funds	GCC	Match Funds	GCC	Match Funds	TOTAL	GCC	Match Funds	TOTAL
PI- Dr. Jena Hamadani	37,039	24,338	56,216	26,772	93,255	51,111	144,366	93,255	51,111	144,366
Co-PI- Dr. Fahmida Tofail	2,701	8,253	4,722	9,078	7,423	17,331	24,753	7,423	17,331	24,753
Co-PI- Dr. Dewan Md Emdadul Haque	16,769	16,505	27,857	18,156	44,626	34,661	79,287	44,626	34,661	79,287
Finance Director	12,690		19,986		32,676		32,676	32,676		32,676
Finance manager	16,925	34,737	27,927	38,211	44,852	72,948	117,800	44,852	72,948	117,800
HR manager	15,885	26,053	26,211	28,658	42,096	54,711	96,807	42,096	54,711	96,807
Head, Regulatory and Legal Affairs	8,779		14,229		23,009		23,009	23,009		23,009
Head, Research Administration	21,432		35,372		56,804		56,804	56,804		56,804
Finance Assisdtant (MCHD)	1,923	10,296	4,231	11,326	6,155	21,622	27,777	6,155	21,622	27,777
Sr. Admn Officer (MCHD)	11,204		18,466		29,670		29,670	29,670		29,670
Office Manager	7,681	9,643	12,169	10,607	19,850	20,249	40,100	19,850	20,249	40,100
Co-I Assistant Scientist	1,683		2,662		4,345		4,345	4,345		4,345
Co-I Assistant Scientist	1,363		2,151		3,514		3,514	3,514		3,514
Liaison member	25,635		42,298		67,933		67,933	67,933		67,933
Co-I (RI-Training and Quality):	12,529	18,010	17,228	19,811	29,757	37,821	67,578	29,757	37,821	67,578
Senior Master trainer	20,659		25,565		46,224		46,224	46,224		46,224
2 Master trainer (Research Investigator)	23,299	28,164	35,239	30,980	58,538	59,144	117,682	58,538	59,144	117,682
Senior Research Officers (Training & Quality)	4,196	9,136	5,769	10,049	9,965	19,185	29,150	9,965	19,185	29,150
Field Research Officers (Training & Quality)	10,073	4,871	18,467	5,358	28,539	10,229	38,768	28,539	10,229	38,768
Field Research Officer	5,036		1,847		6,883		6,883	6,883		6,883
Field Research Assistant	19,729		7,234		26,963		26,963	26,963		26,963

Bayley Tester (FRA)	6,576		6,028		12,605		12,605	12,605		12,605
Senior Field Assistants	7,289		12,027		19,317		19,317	19,317		19,317
Data management Supervisor	10,874		17,942		28,817		28,817	28,817		28,817
Office/Field attendent	5,605		9,248		14,852		14,852	14,852		14,852
Field Organizer (Office)	4,384		7,234		11,618		11,618	11,618		11,618
**Total 1.1 Remuneration - funding recipient's employees**	**311,961**	**190,005**	**458,326**	**209,006**	**770,286**	**399,011**	**1,169,2 97**	**770,286**	**399,011**	**1,169,297**

Budget Justifications

Please provide one page statement justifying the budgeted amount for each major item, including the use of human resources, major equipment, and laboratory services.

Personnel: Dr Jena Hamadani is the project lead. She will dedicate almost 30% of her time to the implementation and full conduct of the project. Dr Fahmida Tofail; the Co-PI of the project will spend 15% of her time. She will help in manual development and visiting Health officials, motivating their staff, analyzing data and drafting manuscripts. Dr. Emdadul Haque will spend 30% of his time to help in implementing intervention and liaison with Govt. Stakeholders. Dr Helen Baker-Henningham, will develop the training curriculum for the Group Intervention and will also help in data analyses and manuscript writing. Prof. Sally Grantham-McGregor will help in planning this scale-up project. She will provide advice on how to go forward and how to trouble-shoot. She will also help in data analyses and manuscript writing. Two Senior Research Investigators will work part time on this project and will be in charge of communicating with the GOB and maintaining a close liaison with the Ministries. There will be 3 master trainers (RIs), who will organize the trainings and observer and coach the health officers. In addition GCC requires Liaison member who will keep close contact with the GoB as well as GCC. The two research investigators will be in charge of test supervision and intervention implementation. There will be1 senior Research Officer and 2 Research Officers responsible for trainings, quality control of intervention. In addition we need 6 junior facilitators who will be working at the field and keep track of all the stimulation activities as well as arrange logistics. We need 4 testers to perform Bayley test and anthropometry twice on 1200 children in the study. One data management supervisor is required for data entry and cleaning. Five Field Organizers are required to maintain liaison with CCs and are required to run errands like delivering letters to the ministries, collecting materials for tests, photocopying questionnaires, etc.

Travel: the PI will travel once every year to UK to discuss the plans with the 2 consultants in that country. The last trip will be to analyze the data and draft manuscripts. Dr. Helen Henningham will visit the field once during the preparatory phase and another time at the beginning of the intervention activities. Some local travelling expenses are required for the staff to move from one village to another and to bring the children for tests to the CCs. We will require some funds to prepare stimulation materials and for printing of books, manuals and questionnaires and arranging training workshops and launching and dissemination seminars. The equipments required are computers, printers, Bayley kits, and anthropometric tools.

Other Support

Describe sources, amount, duration, and grant number of all other research funding currently granted to PI or under consideration.

NA

Biography of the Investigators (1)

Provide biographical data in the following format for all key personnel including the Principal Investigator. Copy the same format for each of them.
Note: Biography of the External Investigators may, however, be submitted in the format as convenient to them..

1. **Name: Dr. Jena D. Hamadani**

2. **Present Position:** Scientist

3. **Educational background**: (last degree and diploma & training relevant to the present research proposal)

Degree	Institution	Year
Ph D in Child Development	Institute of Child Health, University College London	2004
Diploma in Child Health	Bangladesh Institute of Child Health, Dhaka University	1996
MBBS	Rajshahi Medical College	1984
Training on WPPSI-III and MABC	Institute of Child Health, University College London, U.K.	2005
Training on methods of psychosocial stimulation	Tropical Metabolism Research Unit, University of West Indies, Jamaica	2000

4. **Ethics Certification:**

		If Yes		
		Issuing Authority	Registration No	Valid Until
**No** ☐	Yes ☒	NIH	945553	
**No** ☐	Yes ☒	ICH Good Clinical Practice	193335	

Note: If the response is "no", please get certification from CITI or NIH before study initiation and submit a copy to the Committee Coordination Secretariat

5. **List of ongoing research protocols/ activities**

Protocol/ Activity Number	Role in the protocol/ activity (PI, Co-PI, Co-I)	Starting date	End date	Percentage of time
PR-16063	PI	12/1/16	02/28/20	20
PR-17096	PI	09/29/17	03/28/19	30
PR17009	Co-PI	08/01/17	05/08/19	9
PR18033	Co-PI	07/02/18	05/23/19	6
PR18035	Co-PI	07/01/18	06/30/19	5
PR-17064	PI	01/01/19	06/30/20	25

6. **Publications**

Types of publications	Numbers
a. Original scientific papers in peer-review journals	74
b. Peer reviewed articles and book chapters	1
c. Papers in conference proceedings	3
d. Letters, editorials, annotations, and abstracts in peer-reviewed journals	63
e. Working papers	2
f. Monographs	5

7. **Five recent publications including publications relevant to the present research protocol**

7.1. Hamadani, J. D., Mehrin, S. F., Tofail, F., Hasan, M. I., Huda, S. N., Baker-Henningham, H., . . . Grantham-McGregor, S. (2019). Integrating an early childhood development programme into Bangladeshi primary health-care services: an open-label, cluster-randomised controlled trial. *Lancet Glob Health*, *7*(3), e366-e375. doi: 10.1016/s2214-109x(18)30535-7

7.2. Hossain, S. J., Tofail, F., Hasan, M. I., Mehrin, F., Aktar, S., & **Hamadani, J. (2018)**. Gender differences in the quality of psychosocial stimulation in rural Bangladesh homes. *Child Care Health Dev*, *44*(4), 539-544. doi: 10.1111/cch.125687.3.

7.3. Hossain, S. J., Tofail, F., Hasan, M. I., Mehrin, F., Aktar, S., & Hamadani, J. (2018). Gender differences in the quality of psychosocial stimulation in rural Bangladesh homes. *Child Care Health Dev*, *44*(4), 539-544. doi: 10.1111/cch.12568

7.4. Lancaster, G. A., McCray, G., Kariger, P., Dua, T., Titman, A., Chandna, J., . . . Janus, M. (2018). Creation of the WHO Indicators of Infant and Young Child Development (IYCD): metadata synthesis across 10 countries. *BMJ Glob Health, 3*(5), e000747. doi: 10.1136/bmjgh-2018-000747

7.5. Hamadani, J. D., Tofail, F., Huda, S. N., Alam, D. S., Ridout, D. A., Attanasio, O., & Grantham-McGregor, S. M. (2014). Cognitive deficit and poverty in the first 5 years of childhood in Bangladesh. *Pediatrics, 134(4),* e1001-1008. doi: 10.1542/peds.2014-0694

Certificate of Completion

The National Institutes of Health (NIH) Office of Extramural

rch certifies that **Jena Hamadani** successfully completed the NIH

-based training course “Protecting Human Research Participants”.

Certificate of Completion

Date of completion: 07/01/2012

Certification Number: 945553

Biography of the Investigators (2)

Provide biographical data in the following format for all key personnel including the Principal Investigator. Copy the same format for each of them. **Note**: Biography of the External Investigators may, however, be submitted in the format as convenient to them.

Biography of the Investigators

1 **Name: Dr. Fahmida Tofail**

2 **Present Position:** Scientist & Consultant Physician (Registration Number: A 23209)

3 **Educational background**: (last degree and diploma & training relevant to the present research proposal)

	Institution	Year
Post. Doc	Division of Metals & Health, Karolinska Institutet, Stockholm, Sweden,	Continuing
PhD in Child Development	Institute of Child Health, University College London	2006
MBBS	Mymensing Medical College	1992

4. Ethics Certification:

		If Yes		
		Issuing Authority	Registration No	Valid Until
**No** ☐	Yes ☒	National Institutes of Health (NIH)	945557	

5. List of ongoing research protocols/ activities

Protocol/ Activity Number	Role in the protocol/ activity (PI, Co-PI, Co-I)	Starting date	End date	Percentage of time
PR-16037 (RINEW)	Co-I	2016	2018	20
PR-14014	Co-PI	2014	2017	10

PR-15002 (UBS)	PI	2015	2017	9
Act-00806	Co-PI	2016	2017	10
PR-14110 (Neuro)	Co-PI	2014	2017	10

6. Publications

Types of publications	Numbers
a. Original scientific papers in peer-review journals	56
b. Peer reviewed articles and book chapters	3
c. Papers in conference proceedings	
d. Letters, editorials, annotations, and abstracts in peer-reviewed journals	26
e. Working papers	
f. Monographs	1

7. Five recent publications including publications relevant to the present research protocol

i. **Hamadani, J. D.,** Mehrin, S. F., Tofail, F., Hasan, M. I., Huda, S. N., Baker-Henningham, H., . . . Grantham-McGregor, S. (2019). Integrating an early childhood development programme into Bangladeshi primary health-care services: an open-label, cluster-randomised controlled trial. *Lancet Glob Health, 7*(3), e366-e375. doi: 10.1016/s2214-109x(18)30535-7

ii. Hossain, S. J., Tofail, F., Hasan, M. I., Mehrin, F., Aktar, S., & **Hamadani, J. (2018)**. Gender differences in the quality of psychosocial stimulation in rural Bangladesh homes. *Child Care Health Dev, 44*(4), 539-544. doi: 10.1111/cch.125687.3. Hossain, S. J., Tofail, F., Hasan, M. I., Mehrin, F., Aktar, S., & Hamadani, J. (2018). Gender differences in the quality of psychosocial stimulation in rural Bangladesh homes. *Child Care Health Dev, 44*(4), 539-544. doi: 10.1111/cch.12568

iii. **Tofail F.** & Hamadani JD. Prevalence of iron-defciency anaemia among young children in rural Bangladesh Health and Science Bulletin (English) Vol. 8 No. 2 June 2010; pg-1-22 *(IF:KN)*

iv. Hoest C, Seidman JC, Pan W, Ambikapathi R, Kang G, Kosek M, Knobler S, Mason

v. CJ, Miller M; MAL-ED Network Investigators. Evaluating associations between vaccine response and malnutrition, gut function, and enteric infections in the MAL-ED cohort study: methods and challenges. Clin Infect Dis. 2014 Nov 1;59 Suppl 4:S273-9.

vi. Caulfield LE, Bose A, Chandyo RK, Nesamvuni C, de Moraes ML, Turab A, Patil C, Mahfuz M, Ambikapathi R, Ahmed T; MAL-ED Network Investigators. Infant feeding practices, dietary adequacy, and micronutrient status measures in the MAL-ED study. Clin Infect Dis. 2014 Nov 1;59 Suppl 4:S248-54.

Certificate of Completion

The National Institutes of Health (NIH) Office of Extramural Research

certifies that **Fahmida Tofail** successfully completed the NIH Web-based

training course “Protecting Human Research Participants”.

Date of completion: 07/01/2012

Certification Number: 945557

Biography of the Investigators (3)

Provide biographical data in the following format for all key personnel including the Principal Investigator. Copy the same format for each of them. **Note**: Biography of the External Investigators may, however, be submitted in the format as convenient to them..

1. **Name:** Dr. Dewan Md. Emdadul Hoque
2. **Present Position:** Scientist

Biography of the Investigators (4)

Provide biographical data in the following format for all key personnel including the Principal Investigator. Copy the same format for each of them. **Note**: Biography of the External Investigators may, however, be submitted in the format as convenient to them.

1. **Name: Shams El Arifeen**

2. **Present Position:** Senior Scientist and Director
Maternal and Child Health Division, icddr,b

3. **Educational background**: (last degree and diploma& training relevant to the present research proposal)

	Institution	Year
M.B.B.S.	Dhaka Medical College, University of Dhaka, Dhaka Bangladesh	1978-83
M.P.H	Johns Hopkins University School of Hygiene and Public Health, Baltimore, USA	1990-91
DrPH	Johns Hopkins University School of Hygiene and Public Health, Baltimore, USA	1991-97

4. **Ethics Certification:**

		If Yes		
		Issuing Authority	Registration No	Valid Until
**No** ☐	Yes ☒	National Institutes of Health (NIH)		

Note: If the response is “no”, please get certification from CITI or NIH before study initiation and submit a copy to the Committee Coordination Secretariat

5. **List of ongoing research protocols/ activities**

Protocol/ Activity Number	Role in the protocol/ activity (PI, Co-PI, Co-I)	Starting date	End	Percentage of time
Activ. # 00-434	PI	January	December	15%
Pro #2033-024	PI	January	February	5%
PR-10012	PI	January	April	5%
Activ.# 00-454	PI	January	December	41%
Activ. # 00-314	PI	January	February	34%
Activ. #00-442	PI	March	September	5%

6. **Publications**

Types of publications	Numbers
a. Original scientific papers in peer-review journals	210
b. Peer reviewed articles and book chapters	
c. Papers in conference proceedings	
d. Letters, editorials, annotations, and abstracts in peer-reviewed journals	
e. Working papers	
f. Monographs	

7. **Five recent publications including publications relevant to the present research protocol**

7.1. Doi M, Sultana Rekha R, Ahmed S, Okada M, Kumar Roy A, El Arifeen S, Ekström EC, Raqib R, Wagatsuma Y. Association between calcium in cord blood and newborn size in Bangladesh. Br J Nutr. 2011:1-10

7.2. Choudhuri D, Huda T, Theodoratou E, Nair H, Zgaga L, Falconer R, Luksic I, Johnson HL, Zhang JS, El Arifeen S, Nelson CB, Borrow R, Campbell H, Rudan I. An evaluation of emerging vaccines for childhood meningococcal disease. BMC Public Health. 2011;11 Suppl 3:S29. Review

7.3. Catto AG, Zgaga L, Theodoratou E, Huda T, Nair H, El Arifeen S, Rudan I, Duke T, Campbell H. An evaluation of oxygen systems for treatment of childhood pneumonia. BMC Public Health. 2011;11 Suppl 3:S28. Review

7.4. Huda T, Nair H, Theodoratou E, Zgaga L, Fattom A, El Arifeen S, Rubens C, Campbell H, Rudan I. An evaluation of the emerging vaccines and immunotherapy against staphylococcal pneumonia in children. BMC Public Health. 2011;11 Suppl 3:S27. Review

7.5. Webster J, Theodoratou E, Nair H, Seong AC, Zgaga L, Huda T, Johnson HL, Madhi S, Rubens C, Zhang JS, El Arifeen S, Krause R, Jacobs TA, Brooks AW, Campbell H, Rudan I. An evaluation of emerging vaccines for childhood pneumococcal pneumonia. BMC Public Health. 2011;11 Suppl 3:S26. Review

Biography of the Investigators (5)

Provide biographical data in the following format for all key personnel including the Principal Investigator. Copy the same format for each of them. **Note**: Biography of the External Investigators may, however, be submitted in the format as convenient to them.

3. **Name:** Syeda Fardina Mehrin
4. **Present Position:** Research Investigator
5. **Educational background**: (last degree and diploma & training relevant to the present research proposal)

	Institution	Year
Degree	M.Sc in Psychology , University of Dhaka	2000
Degree	BSC in Psychology , University of Dhaka	1999
Training	Advance Biostatistical method and STATA	2016
Training	Advance course on Epidemiology, Research method and SPSS	2016
Training	Scientific Project Management, icddr,b	2014
Training	Exclusive course on “Pathway to Manuscript Writing”, Training and Education Unit (TEU), icddr,b	2009
Training	Bayley-III in India, WPPSI, MABC in UK	
Training	Introductory Course on Epidemiology and Biostatistics at icddr,b	2003

6. **Ethics Certification:**

		If Yes		
		Issuing Authority	Registration No	Valid Until
**No** ☐	Yes ☒	NIH	1335239	

7. List of ongoing research protocols/ activities

Protocol Number	Role in the protocol	Starting date	End date	% of time
PR-16063	Co-I	1/12/2016	28/02/20	50

8. Publications

Types of publications	Numbers
g. Original scientific papers in peer-review journals	12
h. Peer reviewed articles and book chapters	2
i. Papers in conference proceedings	2
j. Letters, editorials, annotations, and abstracts in peer-reviewed journals	20
k. Working papers	
l. Monographs	

9. Five recent publications including publications relevant to the present research protocol

i. Hamadani, J. D., **Mehrin, S. F**., Tofail, F., Hasan, M. I., Huda, S. N., Baker-Henningham, H., . . . Grantham-McGregor, S. (2019). Integrating an early childhood development programme into Bangladeshi primary health-care services: an open-label, cluster-randomised controlled trial. *Lancet Glob Health, 7*(3), e366-e375. doi: 10.1016/s2214-109x(18)30535-7

ii. Hossain, S. J., Tofail, F., Hasan, M. I., **Mehrin, F.,** Aktar, S., & Hamadani, J. (2018). Gender differences in the quality of psychosocial stimulation in rural Bangladesh homes. *Child Care Health Dev, 44*(4), 539-544. doi: 10.1111/cch.125687.3. Hossain, S. J., Tofail, F., Hasan, M. I., Mehrin, F., Aktar, S., & Hamadani, J. (2018). Gender differences in the quality of psychosocial stimulation in rural Bangladesh homes. *Child Care Health Dev, 44*(4), 539-544. doi: 10.1111/cch.12568

iii. Gustin, K., Tofail, F., **Mehrin, F**., Levi, M., Vahter, M., & Kippler, M. (2017). Methylmercury exposure and cognitive abilities and behavior at 10 years of age. *Environ Int*. doi: 10.1016/j.envint.2017.02.004

iv. Tofail F, Hamadani JD, **Mehrin F**, Ridout DA, Huda SN, Grantham-McGregor SM. Psychosocial stimulation benefits development in nonanemic children but not in anemic, iron-deficient children. *Journal of Nutrition* 143:885-893, 2013.

v. Tofail, F., Hamadani, J. D., Ahmed, A. Z., **Mehrin, F.,** Hakim, M., & Huda, S. N. (2012). The mental development and behavior of low-birth-weight Bangladeshi infants from an urban low-income community. *Eur J Clin Nutr, 66*(2), 237-243. doi: 10.1038/ejcn.2011.165

Certificate of Completion

The National Institutes of Health (NIH) Office of Extramural Research certifies that **Syeda Mehrin** successfully completed the NIH Web-based training course “Protecting Human Research Participants”.

Date of completion: 11/27/2013

Certification Number: 1335239

Biography of the Investigators (6)

Provide biographical data in the following format for all key personnel including the Principal Investigator. Copy the same format for each of them. **Note**: Biography of the External Investigators may, however, be submitted in the format as convenient to them.

1. **Name:** Dr. Mohammed Imrul Hasan
2. **Present Position:** Assistant Scientist
3. **Educational background**: (last degree and diploma & training relevant to the present research proposal)

	Institution	Year
**Masters in Public Health**	State University of Bangladesh	**2013**
**MBBS**	Dhaka Medical College, Bangladesh	**2001**
IMCI Clinical Management Training		2005

4. **Ethics Certification:**

		If Yes		
		Issuing Authority	Registration No	Valid Until
**No** ☐	Yes ☒	NIH	1334483	

5. **List of ongoing research protocols/ activities**

Protocol/ Activity Number	Role (PI, Co-PI, Co-I)	Starting date	End date	Percentage of time
PR-16063	Co-I	2016	2020	100

6. **Publications**

Types of publications	Numbers
**a.** Original scientific papers in peer-review journals	08
**b.** Peer reviewed articles and book chapters	
**c.** Papers in conference proceedings	
**d.** Letters, editorials, annotations, and abstracts in peer-reviewed journals	01
**e.** Working papers	
**f.** Monographs	

7. **Five recent publications including publications relevant to the present research protocol**

a. Hamadani, J. D., Mehrin, S. F., Tofail, F., **Hasan, M. I.,** Huda, S. N., Baker-Henningham, H., . . . Grantham-McGregor, S. (2019). Integrating an early childhood development programme into Bangladeshi primary health-care services: an open-label, cluster-randomised controlled trial. *Lancet Glob Health, 7*(3), e366-e375. doi: 10.1016/s2214-109x(18)30535-7

b. **Hasan, M. I**., Hossain, S. J., Braat, S., Dibley, M. J., Fisher, J., Grantham-McGregor, S., ... & Biggs, B. A. (2017). Benefits and risks of Iron interventions in children (BRISC): protocol for a three-arm parallel-group randomised controlled field trial in Bangladesh. BMJ open, 7(11), e018325.

c. Hossain, S. J., Tofail, F., **Hasan, M. I.,** Mehrin, F., Aktar, S., & Hamadani, J. (2018). Gender differences in the quality of psychosocial stimulation in rural Bangladesh homes. *Child Care Health Dev, 44*(4), 539-544. doi: 10.1111/cch.125687.3. Hossain, S. J., Tofail, F., Hasan, M. I., Mehrin, F., Aktar, S., & Hamadani, J. (2018). Gender differences in the quality of psychosocial stimulation in rural Bangladesh homes. *Child Care Health Dev, 44*(4), 539-544. doi: 10.1111/cch.12568

d. Ali, H., Hamadani, J., Mehra, S., Tofail, F., **Hasan, M. I.,** Shaikh, S., . . . Christian, P. (2017). Effect of maternal antenatal and newborn supplementation with vitamin A on cognitive development of school-aged children in rural Bangladesh: a follow-up of a placebo-controlled, randomized trial. *Am J Clin Nutr*. doi: 10.3945/ajcn.116.134478

e. Takeuchi, H., Khan, A. F., Yunus, M., **Hasan, M. I.,** Hawlader, M. D. H., Takanashi, S & Nakahara, S. (2016). Anti-Ascaris immunoglobulin E associated with bronchial hyper-reactivity in 9-year-old rural Bangladeshi children. *Allergology International*, *65*(2), 141-146.

icate of Completion

ational Institutes of Health (NIH) Office of Extramural Research certifies that **mmed Imrul Hasan** successfully completed the NIH Web-based training "Protecting Human Research Participants".

f completion: 11/26/2013

cation Number: 1334483

Biography of the Investigators (7)

Provide biographical data in the following format for all key personnel including the Principal Investigator. Copy the same format for each of them. **Note**: Biography of the External Investigators may, however, be submitted in the format as convenient to them.

1. **Name:** Shamima Shiraji
2. **Present Position:** Research Investigator
3. **Educational background**: (last degree and diploma & training relevant to the present research proposal)

	Institution	Year
Degree	M.Sc in Psychology , University of Dhaka	2002
Degree	BSC in Psychology , University of Dhaka	2000
Training	Advance course on Epidemiology, Research method and SPSS	2016
Training	Basic Course on **Qualitative Research**	2015
Training	Training course on **Information Literacy and Literature Search**	2012
Training	Introductory Course on Epidemiology and Biostatistics at icddr,b	2007
Training	**HIV counselling STI (Sexual Transmitted diseases)** conducted by Family Health International (FHI)	2006

4. **Ethics Certification:**

		If Yes		
		Issuing Authority	Registration No	Valid Until
**No** ☐	Yes ☒	NIH	**2369629**	

Note: If the response is "no", please get certification from CITI or NIH before study initiation and submit a copy to the Committee Coordination Secretariat

5. **List of ongoing research protocols/ activities**

Protocol/ Activity Number	Role in the protocol/ activity (PI, Co-PI, Co-I)	Starting date	End date	Percentage of time

6. **Publications**

Types of publications	Numbers
a. Original scientific papers in peer-review journals	3
b. Peer reviewed articles and book chapters	
c. Papers in conference proceedings	
d. Letters, editorials, annotations, and abstracts in peer-reviewed journals	
e. Working papers	
f. Monographs	

7. Five recent publications including publications relevant to the present research protocol

1. Hamadani JD**,** Tofail F, Hilaly A, Mehrin F, **Shiraji** S, Banu S, S.N. Huda SN. Association of postpartum

maternal morbidities with children's mental, psychomotor and language development in rural Bangladesh. J Health Popul Nutr 2012 Mar;30(2):193-204
2. Hamadani JD, Tofail F, Nermell B, Gardner R, **Shiraji S**, Bottai M, Arifeen SE, Huda SN, Vahter M. Critical windows of exposure for arsenic-associated impairment of cognitive function in pre-school girls and boys: a population-based cohort study. Int J Epidemiol. 2011 Dec;40(6):1593-604.
3. Hamadani JD, Tofail F, Nermell B, Gardner R, **Shiraji S**, Bottai M, Arifeen SE, Huda SN, Vahter M. Critical windows of exposure for arsenic-associated impairment of cognitive function in pre-school girls and boys: a population-based cohort study. Int J Epidemiol. 2011 Dec;40(6):1593-604.
4.

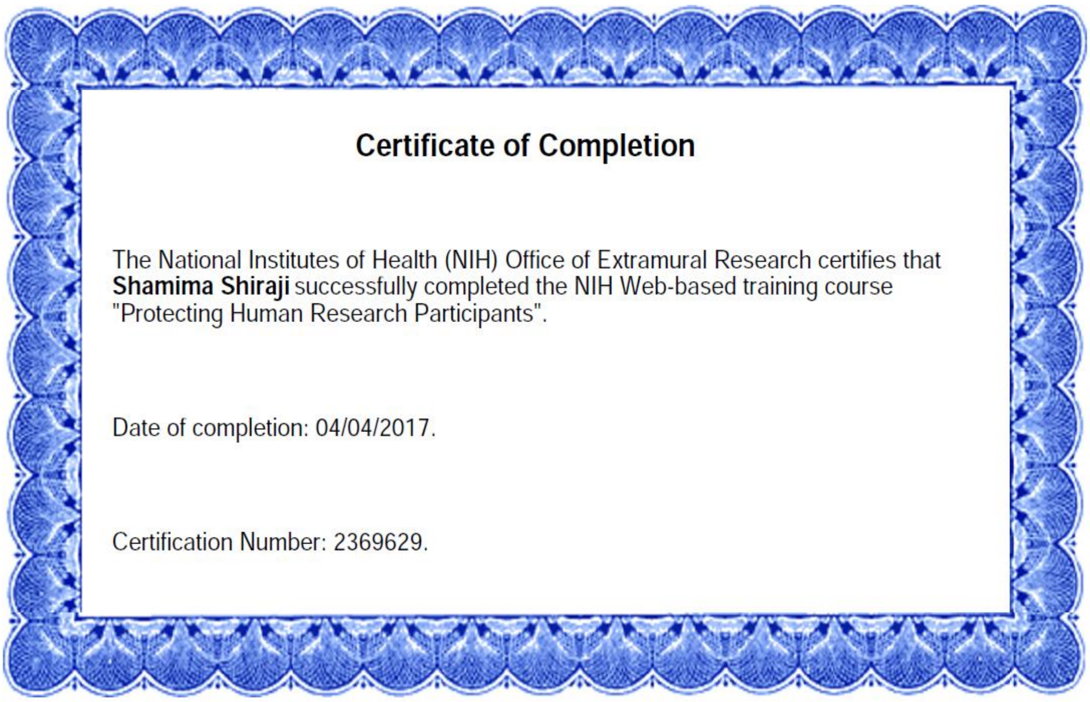

Certificate of Completion

The National Institutes of Health (NIH) Office of Extramural Research certifies that **Shamima Shiraji** successfully completed the NIH Web-based training course "Protecting Human Research Participants".

Date of completion: 04/04/2017.

Certification Number: 2369629.

Biography of the Investigators (8)

Provide biographical data in the following format for all key personnel including the Principal Investigator. Copy the same format for each of them. **Note**: Biography of the External Investigators may, however, be submitted in the format as convenient to them.

1. **Name: Sheikh Jamal Hossain**
2. **Present Position:** Assistant Scientist
3. **Educational background**: (last degree and diploma & training relevant to the present research proposal)

	Institution	Year
Master of Public Health (HP&HE)	NIPSOM, University of Dhaka	2009
Master in Health Economics	Institute of Health Economics, University of Dhaka	2002
Post Graduate Diploma in Health Economics	Institute of Health Economics, University of Dhaka	2001
Scientific Project Management	icddr,b	2014
Training on Result Based Management	icddr,b	2015

4. **Ethics Certification:**

		If Yes		
		Issuing Authority	Registration No	Valid Until
**No** ☐	Yes ☒	NIH	2117415	

5. **List of ongoing research protocols/ activities**

Protocol/ Activity Number	Role (PI, Co-PI, Co-I)	Starting date	End date	Percentage of time
15059	PI	1/7/2015	31/3/2016	100%

6. **Publications**

Types of publications	Numbers
a) Original scientific papers in peer-review journals	1
b) Peer reviewed articles and book chapters	
c) Papers in conference proceedings	

d) Letters, editorials, annotations, and abstracts in peer-reviewed journals	5
e) Working papers	
f) Monographs	1

7. Five recent publications including publications relevant to the present research protocol

I. **Hossain, S. J.,** Tofail, F., Hasan, M. I., Mehrin, F., Aktar, S., & **Hamadani, J. (2018)**. Gender differences in the quality of psychosocial stimulation in rural Bangladesh homes. *Child Care Health Dev, 44*(4), 539-544. doi: 10.1111/cch.125687.3. Hossain, S. J., Tofail, F., Hasan, M. I., Mehrin, F., Aktar, S., & Hamadani, J. (2018). Gender differences in the quality of psychosocial stimulation in rural Bangladesh homes. *Child Care Health Dev, 44*(4), 539-544. doi: 10.1111/cch.12568

II. F Akter, RA Sarker, , SJ Hossain, F Tofail 'Adaptation of Cognitive Behavioral Therapy (CBT) in the cultural perspective of Bangladesh; an integration of quantitative and qualitative approach' (Year:2014) abstract presentation at 'National Public health conference'

III. RA Sarker, F Akter, SJ Hossain, F Tofail. 'Mothers' perception about depressive symptoms: A qualitative study '(Year: 2014) abstract presentation at 'National Public health conference'

Biography of the Investigators (9)

Provide biographical data in the following format for all key personnel including the Principal Investigator. Copy the same format for each of them. **Note**: Biography of the External Investigators may, however, be submitted in the format as convenient to them.

1. **Name:** Afroza Hilaly
2. **Present Position:** Research Investigator
3. **Educational background**: (last degree and diploma & training relevant to the present research proposal)

	Institution	Year
Degree	MSS, Clinical Social Work (CSW), University of Dhaka	2015
Degree	MSS in Sociology and Anthropology , University of Dhaka	2012
Training	Advance course on Epidemiology, Research method and SPSS	2016
Training	Training course on **Information Literacy and Literature Search**	2012
Training	Introductory Course on Epidemiology and Biostatistics at icddr,b	2006

4. **Ethics Certification:**

		If Yes		
		Issuing Authority	Registration No	Valid Until
**No** ☐	Yes ☒	NIH	**491107**	

5. **List of ongoing research protocols/ activities**

Protocol Number	Role in the protocol	Starting date	End date	Percentage of time
PR-16063	Co-I	1/12/2016	28/02/20	100

6. **Publications**

Types of publications	Numbers
Original scientific papers in peer-review journals	2
m. Peer reviewed articles and book chapters	
n. Papers in conference proceedings	
o. Letters, editorials, annotations, and abstracts in peer-reviewed journals	
p. Working papers	
q. Monographs	

7. Five recent publications including publications relevant to the present research protocol

1. Hamadani JD**,** Tofail F, **Hilaly A**, Mehrin F, Shiraji S, Banu S, S.N. Huda SN. Association of postpartum maternal morbidities with children's mental, psychomotor and language development in rural Bangladesh. J Health Popul Nutr 2012 Mar;30(2):193-204

2. Hamadani, J. D., Tofail, F., **Hilaly, A.**, Huda, S. N., Engle, P., & Grantham-McGregor, S. M. (2010). Use of family care indicators and their relationship with child development in Bangladesh. *J Health Popul Nutr, 28*(1), 23-33.

Certificate of Completion

The National Institutes of Health (NIH) Office of Extramural Research certifies that **Afroza Hilaly** successfully completed the NIH Web-based training course "Protecting Human Research Participants".

Date of completion: 08/24/2010

Certification Number: 491107

Biography of the Investigators (10)

1 Name: Dr. Helen Baker Henningham

2 Present Position: Reader in Child Development and Behaviour, School of Psychology, Bangor University, UK

3 Educational background:

	Institution	Year
MSc in Community Disability Studies for Developing Countries	Institute of Child Health, University College London	1998
PhD in International Child Health	Institute of Child Health, University College London	2003
MSc in Child and Adolescent Mental Health	Institute of Psychiatry, Kings College London	2008

4. Ethics Certification:

		If Yes		
		Issuing Authority	Registration No	Valid Until
**No** ☐	Yes ☒	National Institutes of Health (NIH)	1228098	

5. List of ongoing research protocols/ activities

6. Publications: Over 100

7. Five recent publications including publications relevant to the present research protocol

i. Hamadani, J. D., Mehrin, S. F., Tofail, F., Hasan, M. I., Huda, S. N., **Baker-Henningham, H.,** . . . Grantham-McGregor, S. (2019). Integrating an early childhood development programme into Bangladeshi primary health-care services: an open-label, cluster-randomised controlled trial. *Lancet Glob Health, 7*(3), e366-e375. doi: 10.1016/s2214-109x(18)30535-7

II. **Baker-Henningham H**, Vera-Hernandez M, Alderman H, Walker S (2016) Irie Classroom Toolbox: a study protocol for a cluster randomised trial of a universal violence prevention programme in Jamaican preschools. BMJ Open 2016;6:e012166.Doi: 10.1136/bmjopen-2016-012166.

III. Chang SM, Grantham-McGregor S, Powell CA, Vera-Hernandez M, Lopez-Boo F, **Baker-Henningham H,** Walker SP. (2015) Integrating a parenting intervention with routine primary health care: a cluster randomised

i. trial. Pediatrics July 6. pii: peds.2015-0119. [Epub ahead of print PMID: 26148947

IV. **Baker-Henningham H.** The role of early childhood education programmes in the promotion of child and adolescent mental health in low and middle-income countries. International Journal of Epidemiology 2013
i. doi:10.1093/ije/dyt226.

V. **Baker-Henningham H**, Scott S, Walker S. (2012) Reducing child conduct problems and promoting social skills in a developing country: a cluster-randomised controlled trial. British Journal of Psychiatry 201(8):101-8.

VI. Keiling C, **Baker-Henningham H**, Belfer M, Conti G, Ertem I, Omigbodun Y, Rohde L, Srinath S, Ulkuer N,Rahman A. (2011) Global child and adolescent mental health: evidence for action. The Lancet 378(9801):1515-25

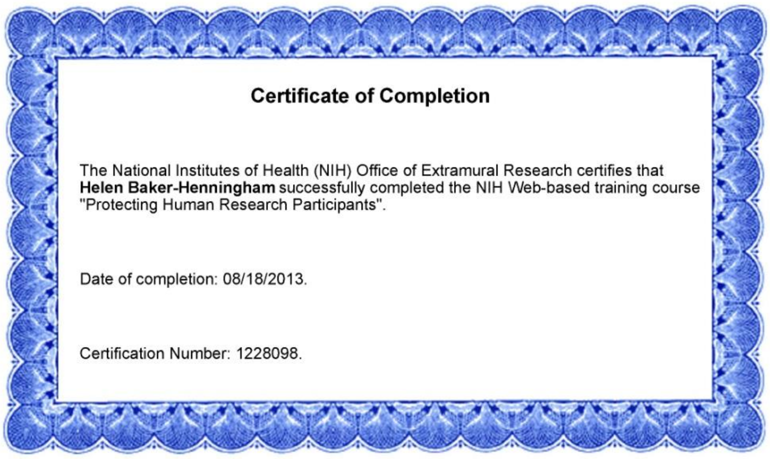

Certificate of Completion

The National Institutes of Health (NIH) Office of Extramural Research certifies that **Helen Baker-Henningham** successfully completed the NIH Web-based training course "Protecting Human Research Participants".

Date of completion: 08/18/2013.

Certification Number: 1228098.

Biography of the Investigators (11)

Provide biographical data in the following format for all key personnel including the Principal Investigator. Copy the same format for each of them. **Note**: Biography of the External Investigators may, however, be submitted in the format as convenient to them.

Name: Sally Grantham-McGregor
Present Position: Emeritus Professor of International Child Health

Qualifications

**Degrees**	**Institution**	**Date**
MB BS	University of London	1963
MD	University of London	1974
Diploma in Public Health	University of the West Indies	1984
FRCP	Royal College of Physicians London	2001

Previous Appointments

2006-10: Emeritus Professor of Child Health and Nutrition, Centre for International Child Health (CICH), Institute of Child Health (ICH), UCL, London, UK

1995-06: Professor of Child Health and Nutrition, (CICH), (ICH), UCL, London, UK

1991-95: Professor in Child Health and Nutrition, Tropical Metabolism Research Unit (TMRU), University of the West Indies, Kingston, Jamaica

1985-91: Reader in Child Health and Nutrition, TMRU

Recent International/National Committees

- Chairman of Steering Committee for the Lancet Series on Child Development in Developing Countries: 2005-07
- Member of the Open Society Institute’s Early Childhood Advisory Board: 2007-present
- Member of Working Group on Iron, the Scientific Advisory Committee on Nutrition (UK government): 2002- 07

Awards

- UNICEF Caribbean Award for Excellence in Child Research, 2007
- Caribbean Health Research Council, special award for "Outstanding achievements in research in nutrition and child development", 2002
- Fellow of the Royal College of Physicians, UK, 2001

Consultancies

I have consulted for: UNICEF, WHO, FAO, UNESCO, WFP, PAHO, Department of Health, South Africa, Ford Foundation USA, Wellcome Trust UK

Academic Supervision:

I have set up research groups on Child Development at the University of the West Indies and the International Center for Diarrhea Research, Bangladesh to look at the effects of nutrition and deprivation on child development and what interventions might help. Both groups are now productive and internationally recognized. I continue to work with them.

Selected Publications in Refereed Journals (from approx 190)

i. Hamadani, J. D., Mehrin, S. F., Tofail, F., Hasan, M. I., Huda, S. N., Baker-Henningham, H., . . . **Grantham-McGregor, S.** (2019). Integrating an early childhood development programme into Bangladeshi primary health-care services: an open-label, cluster-randomised controlled trial. *Lancet Glob Health, 7*(3), e366-e375. doi: 10.1016/s2214-109x(18)30535-7

I. Nahar B, Hamadani JD, Ahmed T, Tofail F, Rahman A, Huda SN, Grantham-McGregor SM. Effects of psychosocial stimulation on growth and development of severely malnourished children in a nutrition unit in Bangladesh. Eur J Clin Nutr. 2009 Jun;63(6):725-31.

II. Hamadani JD, Huda SN, Khatun F, Grantham-McGregor SM. Psychosocial stimulation improves the development of malnourished children in rural Bangladesh. J Nutr. 2006;136 2645-2652

III. Meeks Gardner J, Powell CA, Baker-Henningham H, Walker SP, Cole TJ, Grantham-McGregor SM. Zinc supplementation and psychosocial stimulation: effects on the development of undernourished Jamaican children. Amer J Clin Nutr 82 (2):399-405, 2005

IV. F Tofail, L Persson, S E Arifeen, J D Hamadani , F Mehrin, D Ridout, E Ekström , S N Huda, S M Grantham-McGregor. Effect of prenatal food and micronutrient supplementation on infants' development: A randomised trial from MINIMat study. Amer J Clin Nutr 2008 Mar;87(3):704-11.

V. Walker SP, Chang SM, Younger N, Grantham-McGregor SM. The effect of psychosocial stimulation on cognition and behaviour at 6 years in a cohort of term, low-birthweight Jamaican children. Dev Med Child Neurol. 2010 Jul;52(7):e148-54. Epub 2010 Feb 24

Consent Form

**Protocol No.** PR-19040	**Version No. 1.1**	**Date: 23/04/19**

Project Title: To support the government of Bangladesh to integrate an evidence-based programme of psychosocial stimulation and nutritional counselling into primary health care services and to establish a sustainable organizational structure

Investigator's name: Dr Jena D. Hamadani

Organization: International Centre for Diarrhoeal Disease Research, Bangladesh (icddr,b)

The purpose of this consent form is to provide you with the information to decide if you like to participate in this study.

Purpose of the research

We intend to integrate a programme of psychosocial stimulation into the health system in Bangladesh where the officers at national, district, upazilla and local health and Shishu Academy officers will be trained as trainers and will impart training to the staff of the community clinics who will conduct parenting sessions to groups of malnourished children and their mothers to improve children's development.

Background

As you may know, undernutrition is a major public health problem in Bangladesh which has deleterious effects on future cognitive development. While nutritional intervention is of utmost importance, additional play and stimulation has been shown to benefit children's IQ in the long run. Children with better cognitive functioning usually have higher marks in school and it is possible that in future they will get a better paid job.

Why are you invited to participate in the study?

We are inviting both you and your child to participate in this study, because your child's age is in the range of 6-24 months and your child suffers malnutrition.

Methods and procedures:

If you agree to participate, we may request you to bring your child to the Community Clinic for 1 hour to attend a meeting with the staff of the CC along with other mothers and their children every fortnight for 1 year. The staff of CC will discuss with you about how to raise a child to have better cognitive, language and behaviour development and show you how to make suitable toys for your child from simple household materials at little or no cost. Besides this, if your child is selected for assessment, you will be requested to bring your child to the CC for developmental assessment at the beginning and after one year of the study, where we will assess his/her development by playing with simple toys and asking some questions from you about the activities at home and your knowledge and practices of child rearing. We will also measure yours and your child's nutritional status by measuring his/her height, weight and head circumference and your height and weight. This process may take about 1.5 to 2 hours of your time.

Risk and benefits:

There are no risks involved to you or your child. Your child is very likely to benefit from this study directly, either now or in future. Moreover, results of this research will provide new information on future child development issues and on establishing a sustainable organizational structure in the health system of Bangladesh. In addition, the sessions will include play times and toys, and you and your child will enjoy the sessions. If we find any major problem with your child following our measurements, we will refer your child to appropriate health facilities with the help of the clinic staff where you can seek treatment.

Privacy, anonymity and confidentiality

All information collected from you will remain strictly confidential.

Future use of information

The information collected from this study may be shared with other researchers if needed, but we will strictly maintain your confidentiality and privacy. We or our colleagues, may also contact you in future to follow your child for additional information or measurements.

Right not to participate and withdraw

Your participation is completely voluntary. You can refuse to participate or withdraw from the study at any time, and such a decision will not affect your privilege from attending sessions in CC.

Principle of compensation

There is no monetary compensation for your participation.

Answering your questions/ Contact persons

You are free to ask us questions about the study, if you have any. If you have additional questions later, you may contact Dr. Jena Hamadani at the Dhaka Hospital of icddr,b (*Cholera Hospital, Mohakhali*) or call her at 9827001-10, Ext. 2353 or at 01713093849.

Moreover, you can contact Mr. M. A. Salam Khan, Coordination Manager, Research Administration at 9886498 or 8860523-32. Ext.3206.

If you agree to join the programme, please indicate that by putting your signature or your left thumb impression at the specified space below.

Thank you for your cooperation

______________________________________ ____________________

Signature or left thumb impression of participant Date

______________________________________ ____________________

Signature or left thumb impression of Parent/ Guardian/ Attendant Date

______________________________________ ____________________

Signature or left thumb impression of the witness Date

______________________________________ ____________________

Signature of the PI or his/her representative Date

(NOTE: In case of representative of the PI, she/he shall put her/his full name and designation and then sign)

Consent Form

**Protocol No.** PR-19040	**Version No. 1.10**	**Date: 23.04.29**

গবেষণা প্রকল্পের শিরোনাম:

To support the government of Bangladesh to integrate an evidence-based programme of psychosocial stimulation and nutritional counselling into primary health care services and to establish a sustainable organizational structure

প্রধান গবেষক: ডাঃ জেনা হামাদানী

প্রতিষ্ঠানের নাম: আর্ন্তজাতিক উদরাময় গবেষণা কেন্দ্র, বাংলাদেশ (আই সি ডি ডি আর,বি)

এই সম্মতিপত্রের উদ্দেশ্য হল আপনাকে প্রয়োজনীয় তথ্য প্রদান করা, যে তথ্যগুলো আপনাকে সিদ্ধান্ত নিতে সাহায্য করবে আপনি এই গবেষণায় অংশগ্রহণ করবেন কি না।

গবেষণার উদ্দেশ্য ঃ

আমরা শিশু বিকাশ সংক্রান্তমনো;সামাজিক উদ্দীপনা প্রোগ্রামটি সরকারী কম্যুনিটি ক্লিনিক সেবার সাথে অর্ন্তভূক্ত করতে যাচ্ছি যেখানে জাতীয়, জেলা, উপজেলা এবং স্থানীয় পর্যায়ের ও বাংলাদেশ শিশু একাডেমীর কর্মকর্তাগণকে প্রশিক্ষন দেয়া হবে এবং তারা কম্যুনিটি ক্লিনিকের কর্মীদেরকে প্রশিক্ষিত করবেন, যারা অপুষ্টির শিকার শিশুদের বুদ্ধির বিকাশ উন্নয়নে জন্য কিছুসংখ্যক শিশু ও তাদের মায়েদের সাথে অভিভাবক সভা পরিচালনা করবেন।

ভূমিকা:

হয়তো জেনে থাকবেন যে, শিশুদের অপুষ্টি বাংলাদেশের একটি বড় জনস্বাস্থ্য বিষয়ক সমস্যা যা পরবর্তীতে তাদের মানসিক বিকাশে ক্ষতিকর ভূমিকা রাখে। যদিও পুষ্টি উপাদান অত্যন্ত গুরুত্বপূর্ন, গবেষনায় দেখা গেছে বাচ্চাদের খেলা ও মনোসামাজিক উদ্দিপনা প্রদান পরবর্তীতে বাচ্চার আইকিউ বৃদ্ধিতে ভূমিকা রাখে। ভাল বুদ্ধিসম্পন্ন বাচ্চারা সাধারনত পরবর্তীতে স্কুলে পরীক্ষায় ভাল ফলাফল করে এবং এটাও সম্ভব যে তারা ভবিষ্যতে ভাল চাকুরীও যোগাড় করতে পারে।

আপনাকে কেন এই গবেষণায় অংশগ্রহণের জন্য আহবান করা হচ্ছে?

আমরা আপনাকে এবং আপনার শিশুকে এই গবেষণায় অংশগ্রহণ করার জন্য আমন্ত্রণ করছি যেহেতু আপনার সন্তানের বয়স ৬-২৪ মাসের মধ্যে এবং আপনার বাচ্চা অপুষ্টিতে ভূগছে। ।

কার্যপ্রণালীঃ

আপনি যদি অংশগ্রহন করতে রাজি থাকেন, তাহলে আমরা আপনার । অনুরোধ করতে পারি, বাচ্চা সহ কমিউনিটি ক্লিনিকে ১৫ দিন পর পর আগামী ১ বছরে ধরে এক ঘন্টার জন্য মিটিং এ আসতে, যেখানে ক্লিনিকের কর্মী সহ অন্যান্য মায়েরাও বাচ্চাসহ উপস্হিত থাকবে। কমিউনিটি ক্লিনিক এর নির্ধারিত ব্যক্তি কিভাবে শিশুর লালন পালন করলে শিশুর বুদ্ধি, ভাষা ও আচরণগত বিকাশের উন্নয়ন ঘটানো যায় তা নিয়ে আলোচনা করবে। শিশুদের বুদ্ধির বিকাশ বেশী হলে সাধারনত তারা স্কুলে বেশী নম্বর পায় এবং ভবিষ্যতে তারা ভালো বেতনের চাকরি পেতে পারে । সে আরও দেখাবে অল্প খরচে বা খরচ ছাড়াই কিভাবে আপনার ঘরের জিনিষ দিয়ে আপনার বাচ্চার জন্য উপযোগী খেলনা বানানো যায়। এ ছাড়াও আপনার বাচ্চাটি পরীক্ষার জন্য নির্ধারিত হলে আমরা আপনাকে বাচ্চা নিয়ে গবেষনার শুরুতে এবং এক বছর পর শিশুর বিকাশমূলক পরীক্ষার জন্য কমিউনিটি ক্লিনিকে নিয়ে আসার জন্য অনুরোধ করব। তখন আমরা কিছু সাধারন খেলনা দিয়ে খেলতে দিয়ে আপনার বাচ্চার জ্ঞানীয় বিকাশ পরিমাপ করব এবং আপনি বাড়িতে বাচ্চার সাথে কি কি কাজ করেন, বাচ্চা পালনে আপনার ধারণা ও অভ্যাস সম্পর্কে কিছু প্রশ্ন করব। আমরা আপনার বাচ্চার ওজন, উচ্চতা/দের্ঘ্য, মাথার পরিধি এবং আপনার উচ্চতা ও ওজন পরিমাপের মাধ্যমে পুষ্টির অবস্থা পরিমাপ করব। এই প্রক্রিয়া সম্পন্ন করতে ১.৫-২ ঘন্টা সময় লাগতে পারে।

ঝুঁকি এবং সুবিধাদি ঃ

এই গবেষণায় অংশগ্রহণে আপনার এবং আপনার শিশুর কোন ঝুঁকির সম্ভবনা নেই। এই গবেষণায় অংশগ্রহণ করলে আপনি বাচ্চা স্বাভাবিকভাবেই এই গবেষনা থেকে হয় এখন নয়তো ভবিষ্যতে সরাসরি লাভবান হবে। এছাড়াও এই গবেষনায় প্রাপ্ত ফলাফল ভবিষ্যৎ শিশু বিকাশ সম্পর্কিত বিষয়ে নতুন তথ্য দিবে এবং বাংলাদেশের স্বাস্থ্য ব্যবস্থায় চিরস্থায়ী প্রাতিষ্ঠানিক কাঠামো প্রতীষ্ঠায় সহায়ক হবে। এছাড়াও, যেহেতু সেশনগুলোতে মজার মজার খেলা এবং খেলনা আছে, আপনি এবং আপনার বাচ্চা সেশনটি উপভোগ করবেন। পরিমাপের সময় আমরা আপনার বাচ্চার বড় ধরনের কোন সমস্যা পেলে, কম্যুনিটি ক্লিনিকের কর্মীর সহায়তায় প্রয়োজনীয় চিকিৎসা কোথায় পাবেন সে ব্যাপারে আমরা যথাযথ স্বাস্থ্যকেন্দ্রে যাওয়ার উপদেশ দিব যেখান আপনি সঠিক চিকিৎসা পাবেন।

গোপনীয়তা ঃ গবেষণা চলাকালীন সংগৃহীত সকল তথ্য কঠোর ভাবে গোপন রাখা হবে

তথ্যের ভবিষ্যৎ ব্যবহারঃ

গবেষণালব্ধ তথ্যাদি হয়তোবা অন্যকোন গবেষকদের সাথে উপস্থাপন করা যেতে পারে তবে সেক্ষেত্রে অবশ্যই আপনার নাম পরিচয়ের গোপনীয়তা রক্ষা করা হবে। আপনার বাচ্চার আরো কোন ধরনের পরীক্ষার জন্য আমরা অথবা আমাদের কোন সহকর্মী ভবিষ্যতে আপনার সাথে যোগাযোগ করতে পারেন।

গবেষণায় অংশগ্রহণ ও প্রত্যাহারের অধিকার:

এই গবেষণায় আপনার অংশগ্রহণ সম্পূর্ণ স্বেচ্ছামূলক। আপনি চাইলে গবেষনায় অংশগ্রহন নাও করতে পারেন বা গবেষণা মধ্যবর্তী যে কোন সময়েও নিজেকে প্রত্যাহার করতে পারবেন। এতে কমিউনিটি ক্লিনিকে আপনার সেশনে অংশ গ্রহনে কোন বাধা হবে না।

ক্ষতিপূরণের শর্তাবলীঃ

এ গবেষণায় আপনার অংশগ্রহণের জন্য কোন আর্থিক ক্ষতিপূরণ দেয়া হবে না।

কে এই গবেষণা সম্বন্ধে আপনার প্রশ্নের উত্তর দিতে পারবে?

আপনার যদি কোন প্রশ্ন থাকে আমাদেরকে জিজ্ঞাসা করতে পারেন। আপনি এই ফোন নম্বরে +৮৮০ ২ ৯৮২ ৭০০১ -১০; এক্সটেনশন-২৩৫৩, গবেষণা সংশিণ্টষ্ট কোন প্রশ্ন থাকলে সরাসরি ডাঃ জেনা হামাদানী (কলেরা হাসপাতাল, মহাখালী) জিজ্ঞাসা করতে পারেন।অধিকন্তু আপনি জনাব এম. এ. সালাম খান, কোয়ার্ডিনেসন ম্যানেজার, রিসার্চ এ্যাডমিনেস্ট্রেসন, ফোন-৯৮৮৬৪৯৮ অথবা ৮৮৬০৫২৩-৩২ এক্সটেনশন-৩২০৬ অথবা ০১৭১৩০৯৩৮৪৯ নম্বওে এ যোগাযোগ করতে পারেন।

আপনি যদি এই গবেষণায় আপনি/আপনার শিশুর অংশগ্রহণে রাজি থাকেন তাহলে তা নীচে স্বাক্ষর দিন অথবা বাম হাতের বৃদ্ধাঙ্গুলির ছাপ দিন।

আপনার সহযোগিতার জন্য অনেক ধন্যবাদ।

অংশগ্রহণকারী (নিজের এবং তার শিশুর পক্ষে): ______________________ তারিখঃ ______________________
স্বাক্ষর/বাম বৃদ্ধাঙ্গুলির ছাপ :

সাক্ষীর স্বাক্ষর/বাম বৃদ্ধাঙ্গুলির ছাপঃ ______________________ তারিখঃ ______________________

গবেষকের/প্রতিনিধির স্বাক্ষরঃ ______________________ তারিখঃ ______________________

Detailed Budget for the study titled: ____

Name of Principal Investigator: ___________________________

Protocol Number: ____________

Division: ___________________________

Funding Source: ___________________________

Budget: Director: US$___________; Indirect: US$_________; Total: US$_________

Study period: From:____________ through______________

Strategic Priority Code(s):

**Line Items**	**Budget**										
Payroll and Benefits:	Name of personnel/position	Pay level	% Effort	# of posts	Monthly Rate	Year-1	Year-2	Year-3	Year-4	Year-5	Total amount (US$)
	**Sub-total of Payroll and benefits:**										
Travel and transport											
	**Sub-total of Travel and Transport:**										
Supply and materials											
	**Sub-total of supply and materials:**										
Other contractual											
	**Sub-total of other contractual:**										
**Total direct costs:**											
**Total indirect cost:**											
**Total costs:**											

Check-List

Check-list for Submission of Research Protocol
For Consideration of the Research Review Committee (RRC)
[Please check all appropriate boxes]

1. Has the proposal been reviewed, discussed and cleared by all listed investigators?

   ☒ Yes ☐ No

   If the response is No, please clarify the reasons:

2. Has the proposal been peer-reviewed externally?

   ☐ Yes ☐ No ☒ External Review Exempted

   If the response is 'No' or "External Review Exempted", please explain the reasons: We have requested exemption because the proposal is the scaling up of a previously approved and completed proposal (RR-13099) and there are not many research issue changes from the previous one.

   If the response is "Yes", please indicate if all of their comments have been addressed?

   ☐ Yes (please attach)

   ☐ No (please indicate reason(s)):

3. Has the budget been reviewed and approved by icddr,b's Finance?

   ☐ Yes ☒ No (reason): Part of the budget which comes from the 1st donor (GCC) is approved. The other part, i.e. the matching funds from GoB is sent for approval and will be received soon.

4. Has the Ethics Certificate(s) been attached with the Protocol?

   ☒ Yes ☐ No

   If the answer is 'No', please explain the reasons:

Signature of the Principal Investigator

23.04.2019
Date

Guidelines for Preparing Abstract for ERC

The Ethical Review Committee will not consider any application that does not include an abstract summary. The abstract should summarise the purpose of the study, the methods and procedures to be used, by addressing each of the following items. If an item is not applicable, please note accordingly, describing the reason:

1. Describe the requirements for a 'study population' and explain the rationale for inclusion of special groups in this study population, such as children or groups whose ability to give voluntary informed consents might be compromised.

2. Assess and describe potential risk(s) – physical, psychological, social, legal or other, and also assess their likelihood and seriousness. If research methods are anticipated to involve potential risks, describe alternate methods, if any, which were considered and why they will not be used.

3. Describe procedures for protecting against or minimising potential risks, and an assessment of their likely effectiveness.

4. Include a description of the methods for safeguarding confidentiality and protecting anonymity.

5. When there are potential risks to the participants, or when the privacy of the individual may be affected, the investigators are required to obtain a written informed consent, duly signed by the prospective participants. For minors and individuals with compromised ability to provide a valid consent, informed consent must be obtained from their parents or legal guardians. Describe consent procedures to be followed including how and where informed consent will be obtained.

    a) If signed consent will not be obtained, explain why this requirement should be waived and provide an alternative procedure that would be used.

    b) If information is to be withheld from a participant, provide justification for this course of action.

    c) If there is a potential risk to the participant or privacy of the individual might be affected while applying any particular procedure include a statement in the consent form to clarify whether or not compensation and/or treatment will be available and who will support the costs.

6. If study involves an interview, describe the place and processes, and approximate length of the interview.

7. Assess the potential benefits to be gained or risk the individual participants might be subjected to, and also the benefits that might accrue to the society in general as a result of the planned work. Clarify if and how the benefits outweigh the risks.

8. State if the activity requires the use of records (hospital, medical, birth, death or other), organs, tissues, body fluids, the foetus or the abortus.

    The statement to the potential participants should include information specified in item 2,3,4,5(c) and 7, and also indicate the approximate time they would be required to remain in the activity.

Bayley
Scales of Infant and Toddler Development™
THIRD EDITION

Record Form

Child's name: ____________________

Sex: ☐M ☐F ID #: ____________________

Examiner's name: ____________________

School/Child care program: ____________________

Reason for referral: ____________________

____

Subtest Summary Scores

Subtest	Total Raw Score	Scaled Score	Composite Score	Percentile Rank	Conf. Interval (___%)
**Cognitive** (Cog)					
			Use Table A.5		
**Language** (Lang)					
Receptive Communication (RC)					
Expressive Communication (EC)					
Sum					
			Use Table A.4		
**Motor** (Mot)					
Fine Motor (FM)					
Gross Motor (GM)					
Sum					
			Use Table A.4		
**Social-Emotional** (SE)					
			Use Table A.5		
**Adaptive Behavior**					
*Communication (Com)					
Community Use (CU)					
Functional Pre-Academics (FA)					
Home Living (HL)					
*Health and Safety (HS)					
*Leisure (LS)					
*Self-Care (SC)					
*Self-Direction (SD)					
*Social (Soc)					
*Motor (MO)			(GAC)		
Sum					
			Use Table A.6		

*For children younger than one year, the GAC is calculated using only those skill areas indicated by an asterisk.

Calculate Age and Start Point

	Years	Months	Days
Date Tested			
Date of Birth			
Age			
Age in Months and Days	Years × 12 + months		
Adjustment for Prematurity	Adjust through 24 months		
Adjusted Age			
Start Point	Calculate start point according to chart below		

Age	Start Point
16 days–1 month 15 days	A
1 month 16 days–2 months 15 days	B
2 months 16 days–3 months 15 days	C
3 months 16 days–4 months 15 days	D
4 months 16 days–5 months 15 days	E
5 months 16 days–6 months 15 days	F
6 months 16 days–8 months 30 days	G
9 months 0 days–10 months 30 days	H
11 months 0 days–13 months 15 days	I
13 months 16 days–16 months 15 days	J
16 months 16 days–19 months 15 days	K
19 months 16 days–22 months 15 days	L
22 months 16 days–25 months 15 days	M
25 months 16 days–28 months 15 days	N
28 months 16 days–32 months 30 days	O
33 months 0 days–38 months 30 days	P
39 months 0 days–42 months 15 days	Q

PEARSON

Copyright © 2006, 1993, 1984, 1969 by NCS Pearson, Inc. All rights reserved.
Pearson Executive Office 5601 Green Valley Drive Bloomington, MN 55437
800.627.7271 www.PearsonClinical.com

PsychCorp

26 27 28 29 30 31 32 A B C D E

Product Number: 0154027235

Family Care Indicator Questionnaire

If the information giver (respondent) is other than mother then mention her name:

I want to know about those things with which the child play at home. Please show me those things. These may be home made e.g. home made clay-built toy, doll made up of cloths or toy which is bought & household materials etc. The question should be coded. When mother will show these toys, the question will help her to recall other toys present at home. Code only those toys which the mother can show. Not only the presence of the toys will do, but also these toys should be used for specific play or work mentioned in the following questions.

1. In the last 30 days(Child name) did the child play with any toy that can make music or can be played as musical instrument (e.g. musical instrument or the toys that produce musical sound e.g. plastic mobile as toy, radio as toy, singing doll, tom-tom, pipe etc.) ?
   1= Yes 2= No
2. In the last 30 days did the child (name) play with any toy that can be used for drawing or writing purpose (e.g. picture book for coloring, pencil, pen, chalk, slate or marking / writing with stick in the floor or courtyard etc.
   1= Yes 2= No
3. Is there any picture book suitable for the child (except school book) ?
   1= Yes 2= No
4. In the last 30 days did the child (name) play with anything that disguise himself or take the role of mother, doctor, teacher, actor, doll, plate & cup for acting purpose?
   1= Yes 2= No
5. In the last 30 days(Child name) did the child play with any toy with which he ran about (e.g. ball& bat, rope for jumping, rocking cradle made of rope, a car that can be pulled or pushed etc) ?
   1= Yes 2= No
6. Does the child have any toy with which he can get idea regarding shape (triangular, rectangular, round) & color?
   1= Yes 2= No
7. Does the child have any toy (globular shaped, logo, block) with which tower; house, car etc can be made by placing them one over another or side by side.
   1= Yes 2= No

   Instruction: Answer to the question number 8 & 9 should be in number. If the number is 10 or >10, then the answer should be 10.

8. What is the number of book in your house including school book (except picture book for children
9. What is the number of paper & magazine at your house?

Now I want to know from you regarding some work or play which has been played with the child by you or his father or any senior member of the family, in the last three days.

10. Book was read or picture book/picture/poster was shown to your child by-
    a. Mother b. Father c. other family member who is above 15 years of old
11. Story was told to the child (name) by-
    a. Mother b. Father c. other family member who is above 15 years of old
12. Song, rhyme, religious song was sung to the child (name) by-
    a. Mother b. Father c. other family member who is above 15 years of old
13. Game was played with the child (name) using toys by-
    a. Mother b. Father c. other family member who is above 15 years of old
14. Name of something, counting number & drawing was taught to the child (name) by allocating time for him by-
    a. Mother b. Father c. other family member who is above 15 years of old

Feeling Questionnaire

Sometimes we feel comfortable (console) & sometimes we feel gloomy. Among us there are some people who are always jolly (cheerful) & some always feel disheartened. Now I shall ask you some question regarding your mental status in the last week. We cannot recall the memories of our remote past but can do that of recent past. For this reason, we want to know your mental status of the last 7 days. Last 7 days means, if today is Monday, the period from last Monday morning to yesterday (Sunday) evening & tell me your mental status or how do you feel at that time.

1. In the last week, how many days, did you feel gloomy?
2. In the last week, how many days, did you feel lonely?
3. In the last week, how many days, did you weep?
4. In the last week, how many days, did you enjoy life (cheerful, vivacious or jolly)?
5. In the last week, how many days were you depressed (dejected/ frustrated)?
6. In the last week, how many days, you didn’t get interest doing something or you were not delighted?

Behaviour Rating Scale

১. Approach (প্রাথমিক প্রতিক্রিয়া)

টেস্টারের প্রতি শিশুর প্রারম্ভিক প্রতিক্রিয়া । টেস্টার শিশুর সাথে নিজেকে পরিচিত করে নিয়ে তাকে একটা খেলনা দিয়ে তারপর মায়ের সাথে কথা বলবে। প্রথম ৫-১০ মিনিটের মধ্যে শিশুর প্রতিক্রিয়া তাৎক্ষনিকভাবে করতে হবে, রেটিং পুরো খেলা শেষ করা যাবেনা।

১. এড়িয়ে যাওয়া ঃ তাকে ভীত মনে হয়- মায়ের কোলে লেগে থাকে/ ঘ্যান ঘ্যান বা বিরক্ত করে/ অন্য দিকে তাকায়, নিজেকে গুটিযে নেয।
২. ১ এবং ৩ এর মধ্যে(Between 1and 3) ।
৩. দ্বিধাগ্রস্থ ঃ কিছুটা ভয়/স্পষ্টই উদ্বিগ্ন , টথস্থ/ সাবধান এবং সতর্ক/ খুশী না/হাসে না/ঘ্যান ঘ্যান করেনা/সহজে খেলনা নিয়ে খেলেনা কিন্তু খেলনাটা অল্প ছুঁতে চায়। টেষ্টারের দিকে অল্প সময়ের জন্য তাকাতে পারে(Hesitant: some fear/obviously worried/ wary and watchful/not happy /not smiling/ not fussing/ not readily playing but may be slight touching of toy. May look fleetingly at examiner.)
৪. ৩এবং ৫ এর মধ্যে (Between 3 and 5) ।
৫. গ্রহনীয় ঃ ভয়ের কোন লক্ষণ থাকেনা কিন্তু টেষ্টারের প্রতি সতর্ক থাকে/কিছু দিতে চায়না/টেষ্টারের সাথে কথা বলেনা বা হাসে না / ভীতি ছাড়াই তার দিকে একটু পর পর তাকায়। খেলনা দিয়ে খেলে তবে তার উচ্ছলতা বা উৎসাহ নাই। (Accepting: No sign of fear but aware of examiner /not offering/not vocalising or smiling at examiner / but looking at her from time to time without fear. Plays with toy but not with vigour.)
৬. ৫ এবং ৭ এর মধ্যে (Between 5 and 7) ।
৭. বন্ধুসুলভঃ ভীত নয় । কিছুক্ষন পর টেষ্টারের দিকে তাকিয়ে হাসতে বা কথা বলতে পারা বা তাকে খেলনা দিতে চায়। খেলনা দিয়ে খেলে। (Friendly: Not afraid. May smile or vocalise or offer toy to examiner after a few minutes, plays with toy readily.)
৮. ৭ এবং৯ এর মধ্যে (Between 7 and 9) ।
৯. আমন্ত্রণ জানানো ঃ টেষ্টারকে পুরোপুরি আনন্দের সাথে মেনে নেয়। তাকে হাসি দেয়, তার সাথে কথা বলে বা কাছাকাছি এসে মিশে । স্পষ্টভাবেই খেলনা উপভোগ করে। খেলার প্রবল উৎসাহ দেখাতে পারে । (Inviting: Fully accepts examiner, happily. Interacts with her smiling, vocalising and/ or approaching. Obviously enjoys toy, may show enthusiasm in playing)

২. Adaptability (slow vs quick to warm up)অভিযোজন / পরিবর্তিত অবস্থায় নিজেকে মানিয়ে নেয়ার যোগ্যতা (ধীরগতি বনাম দ্রুততা)):

এই স্কেলটি নির্দেশ করে শিশু কত দ্রুত বা ধীরে্ দক্ষতার সাথে পরিস্থিতি এবং কাজ (নতুন খেলনা, জায়গা, বসা ইত্যাদি) এবং টেস্টারের সাথে খাপ খাওয়াতে পারে। (This scale refers to how quickly or slowly the infant adapts to the examination circumstances and procedures(new tows, place, sitting down, etc) and the examiner)

১। Constantly fearful; never trusting (Bayley-II), Infant remains withdrawn and timid … (TRIB): শিশুটি টেষ্টের পুরো অথবা বেশীর ভাগ সময়ই নির্বিকার বা ভীত থাকতে পারে। পরবর্তী কিছু অংশের টেষ্টের সময় সংকোচ বোধ দেখাতে পারে। কেবল তখনই সামাজিক খেলায় সাড়া দিতে পারে কিন্তু আনন্দ পূর্ণ পারস্পাকির ক্রিয়া শুরু করে না।(Infant remains withdrawn and timid for all or most of the assessment, may show some signs of losing inhibition towards the later_parts of the assessment, only then may respond to social play, but not initiating joyful interaction.)

২। ১ এবং ৩ এ মধ্যে। (Between1 and) ।

৩। Typically fearful; one or two instances of trust (Bayley-II): সাধারনত টেষ্টের ১টা অথবা ২টা অবস্থার উপর আস্থা রাখে অর্ধেক সময়ই নীর্বিকার , সারাক্ষন ভীত এবং অপরিচিত টেষ্টারের প্রতি (Tester) বিরক্ত থাকে। ধীরে ধীরে ভয় কমে আসে এবং আনন্দপূর্ণ পারস্পারিক ক্রিয়া শুরু করে, শব্দ করার মাধ্যমে সামাজিক পারস্পারিক প্রতিক্রিয়া শুরু করতে পারে। টেষ্টের শেষের দিকে টেস্টারকে খেলনা দিতে চাইতে পারে। (Remains withdrawn,timid and bothered by the stranger for about half of the assessment, slowly loser fear, and enters playful interaction, may also initiate social interaction by vocalising,offering toys to the examiner late through the assessment.

৪। ৩ এবং ৫ এর মধ্যে।(Between3 and 5) ।

৫। Fearful one third of the time; trusting the rest of the time (Bayley-II-modified): শিশুর ব্যবহার / আচরণ টেষ্টার দ্বারা প্রভাবিত হতে পারে, কিছু সতর্কতা থাকলেও শিশু টেষ্ট চলাকালীন অবস্থায় টেষ্টারকে প্রথম তৃতীয়াংশের সময়ের মধ্যে মেনে নেয়এবং ধীরে ধীরে সেটা শিথিল হয়ে যায়, পারস্পারিক প্রতিক্রিয়ায় সাড়া দেয়, মা এবং টেষ্টারের সাথে খেলতে শুরু করে। The behaviour is affected by the examiner their is some initial wariness but the infant accepts the assessment situation within the first third of the testing period, relaxes gradually during this first third, opens up to interaction, initiates play with examiner as well as mother.

৬। ৫ এবং ৭ এর মধ্যে। (Between5 and 7) ।

৭। Typically trusting; one or two instances of fear (Bayley-II): সাধারনত আস্থা রাখে। টেষ্টের মধ্যে এক/ দুই বার ভীত থাকে, টেষ্টারকে পুরোপুরিভাবে মেনে নেয়, দেয়া-নেয়া শুরু করে। টেষ্টারের সাথে সম্পৃক্ততা শুরু করে। এতে করে সামাজিক সম্পৃক্ততার জন্য-মায়ের কাছ থেকে সাহায্যের প্রয়োজন খুব কম হয়। Some slight vigilance and restrained behaviour in the first few minutes, but then fully accepts examiner, enters in giving and taking, and takes up interaction with examiner hardly any parental facilitation on the social interaction side is necessary.

৮। ৭ এবং ৯ এর মধ্যে।(Between 7 and 9) ।

৯। Constantly trusting; never fearful (Bayley-II): সারাক্ষন আস্থা রাখে অথবা কখনো ভীত থাকেনা। কোন ভীতি, সাবধানতা বা সংকোচবোধ, ছাড়া পুরো পরিস্থিতি মেনে নেয়। পরীক্ষকের সাথে পারস্পারিক সম্পৃক্ততার জন্য তাৎক্ষনিকভাবে আগ্রহী হয় এবং কোন রকম দেরী বা টেষ্টার বা টেষ্টের পারিপার্শ্বিক পরিস্থিতির সাথে শিশুর গ্রহনযোগ্যতার প্রচেষ্টা ছাড়াই টেষ্ট শুরু করা যায়। (Accepts the entire situation without fear, caution or inhibition of actions, immediately ‘warmed up’ to enter social interaction with examiner and the assessment can start without delay or effort on the examiner’s side in getting infant accepting him/her or the test situation

৩. General Emotional Tone (সামাজিক আবেগীয় প্রকাশ)ঃ

এই স্কেলের মাধ্যমে বোঝা যায় যে টেস্টের সময় শিশু কতটা নিরানন্দ এবং অখুশি বা আনন্দিত এবং হাসিখুশি থাকে।(This scale refers to how unhappy and fussy or cheerful and happy the infant appeared during the examination)

১. শিশুকে টেস্টের পুরো সময় অখুশি দেখা যায়, হতাশ হয়ে যায়,দীর্ঘ সময় ধরে কাঁদে এবং ঘ্যান ঘ্যান করে বা প্রায়ই হু হু শব্দ করে আপত্তি জানাতে পারে।(Child seems unhappy throughout assessment, gets very upset, cries and fusses for long periods or frequently may protest and wail.)
২. ১ এবং ৩ এর মধ্যে্য। (Between 1and 3) ।
৩. প্রায়ই অখুশি থাকে, ঘ্যান ঘ্যান করতে করতে কান্না শুরু করে। আওয়াজ করে কিছুটা আপত্তি জানালেও কিছু কিছু কাজে আনন্দের সাথেই সাড়া দেয়। (At times rather unhappy begins to fuss with cries. Short verbal protest but may respondhappily to some procedures.)
৪. ৩ এবং ৫এর মধ্যে (Between 3 and 5)
৫. মোটমুটি খুশী বা পরিতুষ্ট(১বার বা ২ বার হাসতে পারে এবং মাঝে মধ্যে কোন কাজে ইতিবাচক সাড়া দেয়) মাঝে মধ্যে হতাশ হয়ে পড়লেও সহজভাবে্ই আবার আগের অবস্থায় ফিরে আসে। Moderately happy or contented (may smiles once or twice and positively vocalises occasionally in response to some tasks), may become upset occasionally but recovers fairly easily
৬. ৫ এবং ৭এর মধ্যে(Between 5 and 7) ।
৭. সাধারনভাবেই দেখে মনে হয় খুব খুশি। প্রায়ই উত্তেজনা নিয়ে হাসে। পুরো টেস্টেই খুব অল্প সময়ের জন্য ১বার বা ২ বার অখুশী হয়।(Generally appears to be in a happy state of well being. Smiles often with some excitement. Only becomes briefly unhappy once or twice during the whole assessment.)
৮. ৭ এবং ৯এর মধ্যে (Between 7 and 9) ।
৯. আনন্দে সঞ্চারিত থাকে/আনন্দের বহিঃপ্রকাশ থাকে/অত্যন্ত উদ্দীপ্ত, প্রানবন্ত,অভিব্যাক্তি পরায়ণ,হাস্যরত এবং উৎফুল্ল থাকে। Radiates happiness, highly excited, nothing is upsetting (never becomes upset), animated , expressive, smiling and gleeful .

৪. Attentiveness: মনোযোগের সীমা / পরিধিঃ

এই অধ্যায়টি নির্দেশ করে কতটা সময় শিশু তাকে দেয়া একটি কাজ তাগিদ নিয়ে করতে থাকেএবং বাধা থাকা সত্বেও কাজটি ক্রমাগত অটলভাবে করতে থাকে। (This section refers to the lengh of time that a given activity (task) is pursued by the infant (attention span) and the continuation of an activity in spite of obstacles. The persistence of goal directed effort in at issue.)

১। কাজের প্রতি খুব কম সময়ের জন্য আগ্রহী থাকে। কাজের প্রতি মনোযোগী হওয়ার কোন লক্ষন দেখা যায়না Interested in presented tasks for very brief periods only, no evidence of directed effort or absorption, very short attention.

২। ১ এবং ৩ এর মধ্যে (Between 1 and) ।

৩। কিছুক্ষনের জন্য কাজের প্রতি আগ্রহ থাকে, লক্ষ্যে পৌছার জন্য কিছুটা চেষ্টা করলেও সেটা সহজে- ছেড়ে দেয়, সামান্য পরিমানে/ সীমিত—— মনোযোগ থাকে - বা অল্প সময়ের জন্য কাজটির প্রতি মনোযোগ দেয়, বয়সোপোযোগী কাজটি শেষ করার প্রতি সামান্য আগ্রহ দেখায়। Interested in task for short periods, makes few attempts at goal but easily gives up, little persistence, short attention span or is only fleetingly attending to task, does show little interest in completing age-appropriate tasks.

৪। ৩ এবং ৫ এর মধ্যে (Between 3 and 5) ।

৫। লক্ষ্য অর্জনের জন্য অটলভাবে বারবার কাজটি করতে চেষ্টা করে যেমন কোন জিনিসধরা, বয়সোপোযোগী সমস্যা সমাধান, মাঝামাঝি মনোযেগের সীমা এবং প্রথম প্রচেষ্টায় কাজটি করতে ব্যর্থ হলেও সেটা চালিযে যাওয়া । Makes fairly persistent efforts towards a goal or repeated attempts to achieve a goal (e.g. to attain an object, solve an age- appropriate problems),moderate attention span, and continues task even if failed first attempt.

৬। ৫ এবং ৭ এর মধ্যে।(Between5and 7) ।

৭। লক্ষ্য অর্জন বা সমস্যা সমাধানে ক্রমাগত চেষ্টা করে, দীর্ঘসময় মনোযোগী, অনেকবার ব্যর্থ হলেও সেটা চালিয়ে যেতে থাকে। কাজের সাথে বা লক্ষ্যের সাথে মিশে যাওয়ার তাগিদ থাকে। Persistent efforts to reach goal or solve problem, long periods of attention, attempts task repeatedly even if has failed a number of times, absorbed in the task.

৮। ৭ এবং ৯ এর মধ্যে (Between 7 and 9) ।

৯। দীর্ঘসময় ধরে এবং কাজের প্রতি নিমগ্ন থাকে/ মিশে যায়, এমনকি যদি নতুন কোন কাজ করতে দেখাা যায়, ঐ কাজটি শেষ করার জন্য পরে আবার সে আগের কাজে ফিরে আসে। Very long periods and continued absorption in a task, very persistent, tries to carry on to complete a task, even when new task introduced, may come back to a task after assessment.

৫. Robustness and Endurance - বলিষ্ঠভাব এবং সহনশীলতা

পুরো টেষ্টের সময়টিতে শিশুর ধৈর্য্য রাখার ব্যাপারে এই স্কোরটি নির্দেশ করে। কিছু কিছু শিশু মধ্যমাত্রার আগ্রহ নিয়ে কাজটি শুরু করলেও দ্রুত ক্লান্ত হয়ে পড়ে। তারা খুব দ্রুতই নিষ্কর্ম হয়ে পড়ে, ~~কারন~~ তারা কাজের প্রতি অনাগ্রহ দেখায়, উদ্যমহীন বা খুব বেশি উদ্যমী । অন্য শিশুরা পুরো টেষ্টের সময় তাদের আগ্রহ সচল রাখে। তারা বলিষ্ঠ এবং সহনশীল থাকে এবং টেস্টটি তাদেরকে ক্লান্ত করেনা। টেষ্টার কতটা সহজে শিশুকে টেষ্ট করাতে পারে সেটা হচ্ছে বলিষ্ঠতার চিহ্ন।

This scale refers to the energy/resources available to the infant during the whole testing period. Some infants starts start with moderate energy but tire quickly. They become quickly unavailable because they show low level of functioning, lethargy/or hyper arousal. Other infants mobilize energy/resources during the whole examination. They are robust and have endurance and the test does not seem to tax them. The ease with which the examiner can proceed in the course of the examination can be an index of this robustness.

১। সহজেই ক্লান্ত হয়, আগ্রহ / শক্তি খুব কম থাকে, খুব তাড়াতাড়ি কাজের নিম্নমাত্রার প্রতি ফিরে আসে, প্রারম্ভিক সম্পর্কের ক্ষেত্রে খুব নাজুক এবং কোমল হয়। একটা সেশনে টেষ্টটি সম্পন্ন করা যায় না। (Tires easily, very little energy resources, very quickly regresses to lower levels of functioning, very fragile and delicate in interaction, assessment cannot be completed in one session.)

২। ১ এবং ৩ এর মধ্যে।(Between 1 and 3) ।

৩। টেষ্টটির প্রথমার্ধে যথেষ্ঠ ক্লান্তি নাজুকতা থাকে। শিশুটি অস্থির হয়ে পরে তবে দীর্ঘ সময় ধরে তাকে বিশ্রাম দিলে এবং সময় নিয়ে টেষ্ট করলে টেষ্টটি শেষ করা যেতে পারে।(Considerable exhaustion and fragility starting in the first half of the assessment period ; infant grows restless but with prolonged periods out and slowed timing , the assessment can be completed.)

৪। ৩ এবং ৫ এর মধ্যে (Between 3 and 5)| ।

৫। বেশীরভাগ টেষ্টের জন্য পর্যাপ্ত কর্মশক্তির উদ্দিপনা থাকে। শুধুমাত্র টেষ্টের শেষের দিকে ক্লান্ত বা অস্থির হয়ে যায় । শিশুর কিছুটা সময়ের জন্য বিশ্রামের প্রয়োজনও হতে পারে। (Adequate energy resources for most of the assessment, only tires or gets restless towards the end of the testing , infant may need few brief periods of time out)

৬। ৫ এবং ৭ এর মধ্যে(Between 5 and 7) ।

৭। শিশুটি টেষ্টের পুরো সময়টাতে ভাল ভাবে কাজ করতে পারে । ভালো কর্ম শক্তি থাকে। একবার হয়তো কিছুক্ষনের জন্য বিশ্রামের সময় লাগতে পারে (Holds up well throughout testing period , good energy resources , may only need one brief period of time out)

৮। ৭ এবং ৯ এর মধ্যে (Between7 and 9) ।

৯। খুব বলিষ্ঠ, কর্মদ্যোগ ভাল, ধারাবাহিকভাবে ভাল করে এবং আগ্রহ নিয়ে সাড়া দেয়, এমনকি কঠিন কাজের জন্য দীর্ঘ সময়ের মধ্যেও সাড়া দেয়। (Very robust , good energy resources, continues to respond well and with interest , even during prolonged testing at difficult levels.)

৬. Cooperativeness ঃ সহযোগীতা

এই স্কেলটির মাধ্যমে বোঝা যায় যে,শিশু কতটা টেস্টারের সাথে সহযোগীতা করে এবং তার কথা শুনে। This is a measure of how well the infant co-operates with the examiner and complies with her requests.

১. .টেস্ট সম্পর্কিত সব রকমের নির্দেশ এবং অনুরোধ প্রতিরোধ করে বা করতে চায়না, খুব বেশী বাধা দেয় এবং অসহযোগী। Resists all suggestions or requests, which are assessment related, very resisting and uncooperative.
২. ১ এবং ৩ এর মধ্যে (Between 1 and 3) ।
৩. শুরুতেই অনেক নির্দিষ্ট টেস্ট আইটেমকে ”না” বলে বা প্রতিরোধ করে অথবা সেসনের কিছু অংশে সহযোগীতা করতে প্রত্যাখ্যান করে যেমন শুরুতে বা শেষের দিকে) Refuses or resists several specific examinations initially or refuses to co-operate during part of the session (e.g. initially or towards the end).
৪. ৩ এবং ৫ এর মধ্যে(*Between 3 and 5*)
৫. সেসনটি বা পরিবেশটি মেনে নেয়,টেস্টারের প্রতি সহযোগীও নয় আবার তার কথা স্থাপনে প্রতিরোধও করেনা। মাঝে মধ্যে ”না” বললেও আবারও কাজটি করে। Accepts the assessment or situation, neither cooperative nor resistant in relation to examiner, may occasionally say" No" but will conform
৬. ৫ এবং ৭ এর মধ্যে (Between 5 and 7)
৭. টেস্টারের সাথে পারস্পরিক সম্পর্কটাকে উপভোগ করে বলে মনে হয়, বেশীর ভাগ সময়ই কাজে অংশগ্রহন করেখুশী থাকে।. Seems to enjoy the interaction with the examiner, is happy to participate most of the time.
৮. ৭ এবং ৯ এর মধ্যে(Between 7 and 9)
৯. সেসনটা উপভোগ করে সব কথা শুনে সহজভাবে টেস্টারের কৌশল মেনে নেয়। Enjoys the session and always complies, readily accepts the examiner's manipulation.

৭. Vocalisation (বাচনভঙ্গি)

বাচনভঙ্গি নির্দেশ করে কান্না ছাড়া উচ্চারিত শব্দ অথবা কান্নার মধ্যেও বলা পরিচিত শব্দ। কুজন করা,আধো আধো কথা বলা,ব্যঞ্জনশব্দ বা কথা হতে পারে। যত ধরনের কান্নাই করুক , বিশিষ্ট ভাবে কান্না গ্রহণযোগ্য নয়।Vocalisations refer to non- crying utterances or to recognisable utterances embedded in crying. These may be cooing, babbling, consonant sounds or words. Crying per se, no matter how varied, does not qualify

১. সুস্পষ্টভাবেই নিরব, ১টা বা ২টা শব্দ করে/ বলে(Definitely quiet, 1 or 2 vocalisations).
২. ১ এবং ৩এর মধ্যে (Between 1 and 3).
৩. খুব কম কথা বলে বা শব্দ করে এবং অল্প সময়ের জন্য(Few vocalisations and of short duration)
৪. ৩ এবং ৫ এর মধ্যে(Between 3 and 5).
৫. কার্যক্রমের অংশ হিসাবেই কথা বলে কিন্তু এতটা বিরতি নিয়ে বলে যে সেইটা কোন উত্তেজনা বা অহেতুক কথা বলার মত নয়। Vocalisations occur as part of the activities but too intermittent to constitute vocal excitement, chatter or the like.
৬. ৫ এবং ৭এর মধ্যে(Between 5 and 7).
৭. শব্দ করা বা কথা কলা নির্দিষ্টভাবেই শিশুর কার্যক্রমের একটি অংশ ঃ শিশু কথা বলার বা শব্দ করার জন্যই কথা বলে বা শব্দ করে। Vocalisations constitute an obvious part of the infant's activity: infant vocalises for the sake of vocalising.
৮. ৭ এবং ৯এর মধ্যে (Between 7 and 9).
৯. অতিরিক্ত কথা বলে/ শব্দ করে,কথার মধ্যে প্রচন্ড উত্তেজনা থাকে। (Excessive vocalisations, high vocal excitement)

৮. বিভিন্ন জিনিষ অথবা চারিপাশের পরিবেশের প্রতি অনুসন্ধান (Exploration of objects and/or surroundings):

বাচ্চা দৃষ্টি, শ্রবণ এবং স্পর্শের মাধ্যমে কতটা সক্রিয়ভাবে জিনিষ অথবা চারিপাশের পরিবেশের মধ্যে নতুন কিছু খুঁজে অনুসন্ধান করে। The degree to which the child actively seeks out new aspects of objects or the environment, including the child's visual, auditory and tactile exploration.

১. অনুসন্ধান ছাড়া (No exploration): বাচ্চা একেবারেই খেলার সামগ্রী গুলো অথবা কোনো জিনিস হাত দিয়ে স্পর্শ করে না অথবা নাড়াচারা করে না। চারিপাশের শব্দের প্রতি অনুসন্ধান করে না অথবা সাড়া দেয় না। (Child does not touch or manipulate the objects at all. No exploration of the sounds in the environment.)

২. ১ ও ৩ এর মধ্যে। (Between 1 and 3).

৩. দুই-এক বার অনুসন্ধান করতে দেখা যায়। (One or two instances of exploration): বাচ্চা খেলনা সামগ্রী দুই-একবার নাড়াচাড়া করতে পারে। জিনিষ গুলো দেখা এবং নাড়াচাড়া করার জন্য বাচ্চাকে উৎসাহ দিতে হতে পারে। : Child may manipulate the objects for a couple of times. May need encouragement to look or touch the object.

৪. ৩ এবং ৫ এর মধ্যে(Between 3 and 5).

৫. মাঝারি ধরনের অনুসন্ধান (Moderate exploration): বাচ্চা কয়েকবার জিনিষ গুলো নাড়াচাড়া করে, কখনো কখনো তারা শব্দ কোথা থেকে আসছে এটা অনুসন্ধান করার চেষ্টা করে, টেস্টিং রুমের চারিদিক ঘুরে ঘুরে দেখে এবং নতুন পরিবেশটিকে আবিষ্কার করে (৩-৫ বার) । Child manipulates the objects a number of times, sometimes tries to explore where the sounds are coming from, moves around the testing room and explores the new environment (3-5 times).

৬. ৫ এবং ৭ এর মধ্যে(Between 5 and 7).

৭. অনেক অনুসন্ধান (Much exploration): বাচ্চা বার বার জিনিষগুলো নাড়াচাড়া করে, শব্দ কোথা থেকে আসছে সেটা অনুসন্ধান করার চেষ্টা করে অথবা টেস্টিং রুমের চারিদিকে ঘুরে ঘুরে দেখে এবং পরিবেশটিকে আবিষ্কার করে (≥ ৬ বার) । Child manipulates the objects frequently, tries to explore where the sounds are coming from or moves around the testing room and explores the new environment several times (≥6 times).

৮. ৭ এবং ৯ এর মধ্যে (Between 7 and 9

৯. একটানা অনুসন্ধান (Constant exploration): বাচ্চা একটানা জিনিষগুলো নাড়াচাড়া এবং অনুসন্ধান করে, শব্দ কোথা থেকে আসছে সেটা অনুসন্ধান করার চেষ্টা করে অথবা টেস্টিং রুমের চারিদিকে ঘুরে ঘুরে দেখে এবং নতুন পরিবেশটিকে সারাক্ষন আবিষ্কার করতে থাকে। Child constantly manipulates and explores the objects, tries to explore where the sounds are coming from or moves around the testing room and explores the new environment continuously

৯. খেলা/কাজের প্রতি উদ্যম (Enthusiasm towards task): বাচ্চা যে মাত্রায় খেলনাগুলোর অথবা কাজের প্রতি গভীর মনোযোগ, অনেক উৎসাহ অথবা খুশি প্রদর্শন করে। The degree to which the child exhibits deep concentration, coupled with excitement or delight, in the materials or tasks.

১. একেবারে অবসন্ন, কাজগুলো চালিয়ে যাবে তবে কাজগুলোর প্রতি কোনো বিশেষ আগ্রহ নেই।(Consistently unenthusiastic; no particular interest beyond attending to the tasks.)

২. ১ ও ৩ এর মাঝে(Between 1 and 3).

৩. সাধারণত অবসন্ন : হঠাৎ এক-দুইবার উদ্যমী। (Typically unenthusiastic; enthusiastic in one or two instances.)

৪. ৩ ও ৫ এর মাঝে(Between 3 and 5).

৫. খেলা/কাজের অর্ধেক সময় অবসন্ন।(Unenthusiastic half the time)

৬. ৫ ও ৭ এর মধ্যে(Between 5 and 7).

৭. সাধারনত উদ্যমী, এক-দুইবার অবসন্ন ।(Typically enthusiastic; unenthusiastic in one or two instances)

৮. ৭ ও ৯ এর মধ্যে (Between 7 and 9).

৯. সারাক্ষন একাগ্রতার সাথে উদ্যমী। (Consistently enthusiastic)

সেকশন C ঃ Family Care Indicator Questionnaire

তথ্য প্রদানকারী মা ব্যতিত অন্যকেউ হলে তার নাম:

"আমি সে সমস্ত জিনিস সম্পর্কে জানতে আগ্রহী যা দিয়ে (নাম) বাড়িতে খেলা করে।" দয়া করে আমাকে সেগুলো দেখাবেন। খেলনাগুলো বাড়ীতে তৈরী, যেমনঃ ঘরের তৈরী মাটির খেলনা, কাপড়ের তৈরী পুতুল অথবা কেনা খেলনা এবং বাড়ির জিনিসপত্র ইত্যাদিও হতে পারে।

প্রশ্নগুলো কোড করতে হবে।মা যখন খেলনাগুলো দেখাবে, প্রশ্নগুলো মাকে বাড়িতে আছে এমন অন্যান্য খেলনার কথাও মনে করিয়ে দিবে।যে সমস্ত খেলনা মা দেখাতে পারবে শুধু সেগুলোই কোড করুন।শুধু খেলনা থাকলেই হবে না, সেই খেলনা দিয়ে নির্দিষ্ট প্রশ্নের খেলা খেলতে হবে বা নির্দিষ্ট প্রশ্নের কাজের জন্য ব্যবহার করতে হবে

১. গত ৩০ দিনে (নাম) এমন কোন খেলনা দিয়ে খেলেছে যাতে বাজনা হয় বা বাজানো যায়, (যেমন বাদ্যযন্ত্র অথবা যে সব খেলনা শব্দ তৈরী করে, যেমন; বাচ্চার খেলার জন্য প্লাস্টিকের মোবাইল, খেলনা রেডিও, গান গাওয়ার পুতুল, ঢোল, বাঁশি, ইত্যাদি)? ১= হ্যাঁ,০= না |__|
২. গত ৩০ দিনে (নাম) আঁকাআঁকি বা লেখা যায় এমন জিনিস দিয়ে খেলেছে (যেমন: রং করার জন্যে ছবির বই, পেন্সিল, কলম, চক,শ্লেট, লেখা অথবা কাঠি দিয়ে উঠানের মাটিতে/ ঘরের মেঝেতে দাগাদাগি, ইত্যাদি)? ১= হ্যাঁ, ০= না |__|
৩. বাচ্চাদের উপযোগী কোন ছবির বই আছে (স্কুল বই ছাড়া)? ১= হ্যাঁ, ০= না |__|

৪. গত ৩০ দিনে (নাম) এমন কোনো জিনিস দিয়ে কোন কিছু সেজে বা অভিনয় করে খেলেছে, যেমন মা, ডাক্তার, শিক্ষক, নায়ক, পুতুল, খাওয়ার জন্য প্লেট এবং কাপ, ইত্যাদি? ১= হ্যাঁ,০= না |__|

৫. গত ৩০ দিনে (নাম) ছুটাছুটি করে খেলতে পারে এমন কোনো খেলনা দিয়ে খেলেছে (যেমন বল, দড়ি লাফানো, ব্যাট, দড়ি দিয়ে বানানো দোলনা, টানা বা ঠেলা দেওয়া গাড়ী, ইত্যাদি) ? ১= হ্যাঁ, ০= না |__|

৬. বাচ্চার এমন কোন খেলনা আছে যা দিয়ে বিভিন্ন আকৃতি (তিনকোণা, চারকোণা, গোল) এবং রং শেখা যায় ? ১= হ্যাঁ, ০= না |__|

৭. বাচ্চার এমন কোন খেলনা আছে (যেমন-গুটি,লেগো, ব্লক) যা একটার উপর আরেকটা রেখে, বা পাশাপাশি রেখে টাওয়ার,ঘর, গাড়ী ইত্যাদি বানানো যায় ? ১= হ্যাঁ, ০= না |__|

নির্দেশনা: ৮ ও ৯ নং প্রশ্নের উত্তর সংখ্যায় লিখুন। সংখ্যাটি ১০ বা ১০ এর বেশী হলে ১০ লিখুন

৮. বাড়িতে স্কুল বই সহ কয়টি বই আছে (বাচ্চাদের ছবির বই ছাড়া) ? |__|__|

৯. বাড়িতে কয়টি ম্যাগাজিন এবং পেপার আছে? |__|__|

এখন আপনার কাছে কিছু কাজ বা খেলার কথা জানতে চাইব যা আপনি অথবা বাবা বা বাড়ীর বড় কেউ বাচ্চার সাথে গত ৩ দিনে করেছে কিনা জানাবেন?

১০. বই পড়ে শুনিয়েছেন,ছবির বই /ছবি / পোষ্টার দেখিয়েছেন-

১০.ক মা |__| ১০.খ বাবা |__| ১০.গ পরিবারের অন্যকেউ যার বয়স ১৫ বছরের উপরে |__|

১১. বাচ্চাকে (নাম) গল্প বলেছেন-

১১.ক মা |__| ১১.খ বাবা |__| ১১.গ পরিবারের অন্যকেউ যার বয়স ১৫ বছরের উপরে |__|

১২. গান / ছড়া গান,গজল গেয়েছেন -

১২.ক মা |__| ১২.খ বাবা |__| ১২.গ পরিবারের অন্যকেউ যার বয়স ১৫ বছরের উপরে |__|

১৩. বাচ্চার (নাম) সাথে খেলনা দিয়ে খেলেছেন-

১৩.ক মা |__| ১৩.খ বাবা |__| ১৩.গ পরিবারের অন্যকেউ যার বয়স ১৫ বছরের উপরে |__|

১৪. বাচ্চাকে (নাম) সময় দিয়ে কোন কিছুর নাম, গনণা এবং আঁকাআকি শিখিয়েছেন-

১৪.ক মা |__| ১৪.খ বাবা |__| ১৪.গ পরিবারের অন্যকেউ যার বয়স ১৫ বছরের উপরে |__|
